# MDM2 E3 ligase-mediated ubiquitination and degradation of HDAC1 in vascular calcification

**DOI:** 10.1038/ncomms10492

**Published:** 2016-02-01

**Authors:** Duk-Hwa Kwon, Gwang Hyeon Eom, Jeong Hyeon Ko, Sera Shin, Hosouk Joung, Nakwon Choe, Yoon Seok Nam, Hyun-Ki Min, Taewon Kook, Somy Yoon, Wanseok Kang, Yong Sook Kim, Hyung Seok Kim, Hyuck Choi, Jeong-Tae Koh, Nacksung Kim, Youngkeun Ahn, Hyun-Jai Cho, In-Kyu Lee, Dong Ho Park, Kyoungho Suk, Sang Beom Seo, Erin R. Wissing, Susan M. Mendrysa, Kwang-Il Nam, Hyun Kook

**Affiliations:** 1 Department of Pharmacology and Medical Research Center for Gene Regulation, Chonnam National University Medical School, 5 Hak-dong, Dong-ku, Gwangju 501-746, Republic of Korea; 2 Department of Cardiology, Chonnam University Hospital, Gwangju 501-757, Republic of Korea; 3 Department of Forensic Medicine, Chonnam National University Medical School, 5 Hak-dong, Dong-ku, Gwangju 501-746, Republic of Korea; 4 Department of Pharmacology and Dental Therapeutics, Research Center for Biomineralization Disorders, School of Dentistry, Chonnam National University, Gwangju 500-747, Republic of Korea; 5 Division of Cardiology, Department of Internal Medicine, Seoul National University Hospital, Seoul 110-744, Republic of Korea; 6 Department of Internal Medicine, Kyungpook National University School of Medicine, Daegu 700-721, Republic of Korea; 7 Department of Ophthalmology, Kyungpook National University School of Medicine, Daegu 700-721, Republic of Korea; 8 Department of Pharmacology, Brain Science & Engineering Institute, Kyungpook National University School of Medicine, Daegu 700-721, Republic of Korea; 9 Department of Life Science, Chung-Ang University, Seoul 156-756, Republic of Korea; 10 Department of Basic Medical Sciences, College of Veterinary Medicine, Purdue University, West Lafayette, IN 47907, USA; 11 Departments of Anatomy, Chonnam National University Medical School, 5 Hak-dong, Dong-ku, Gwangju 501-746, Republic of Korea

## Abstract

Vascular calcification (VC) is often associated with cardiovascular and metabolic diseases. However, the molecular mechanisms linking VC to these diseases have yet to be elucidated. Here we report that MDM2-induced ubiquitination of histone deacetylase 1 (HDAC1) mediates VC. Loss of HDAC1 activity via either chemical inhibitor or genetic ablation enhances VC. HDAC1 protein, but not mRNA, is reduced in cell and animal calcification models and in human calcified coronary artery. Under calcification-inducing conditions, proteasomal degradation of HDAC1 precedes VC and it is mediated by MDM2 E3 ubiquitin ligase that initiates HDAC1 K74 ubiquitination. Overexpression of MDM2 enhances VC, whereas loss of MDM2 blunts it. Decoy peptide spanning HDAC1 K74 and RG 7112, an MDM2 inhibitor, prevent VC *in vivo* and *in vitro*. These results uncover a previously unappreciated ubiquitination pathway and suggest MDM2-mediated HDAC1 ubiquitination as a new therapeutic target in VC.

## Abstract (web summary)

Vascular calcification (VC) increases morbidity and mortality in cardiovascular and metabolic diseases. Here, Kwon *et al.* show that calcification stimuli induce MDM2- mediated ubiquitination and proteasomal degradation of HDAC1, suggesting a possible therapeutic strategy for treatment of VC patients.

## Graphical abstract

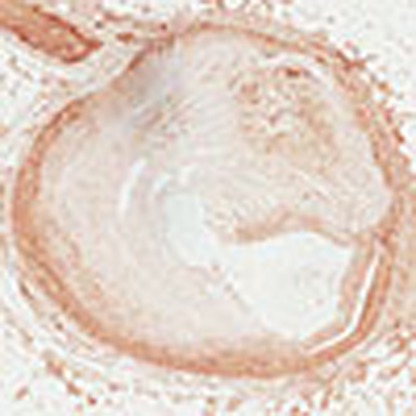

Vascular calcification (VC), a deposition of calcium phosphate in arteries, heart valves and cardiac muscle, often results in the development of atherosclerotic intimal injury and in a rapid rise of stiffness of blood vessels. As a concomitant adverse effect of diabetes and chronic renal failure, VC is often the cause of high morbidity and mortality associated with these chronic diseases^1^. Indeed, VC is closely related to the increase in risk of myocardial infarction and dissection after angioplasty^2^. Despite its clinical implications, the molecular mechanisms of VC have yet to be elucidated. Recent advances utilizing genetically modified mice have shown that VC is an active process rather than a degenerative, passive process at the end stage of vascular diseases^3^. For example, knockout (KO) of matrix Gla protein^4^, KLOTHO^5^,^6^, carbonic anhydrase II^7^, desmin^8^ or osteoprotegerin^9^ in mice elicits marked enhancement of VC, which suggests that VC is in fact actively regulated by vascular smooth muscle acquiring osteoblast-like properties.

Many researchers have reported that histone deacetylase (HDAC) inhibitors (HDACi) are beneficial in regression of tumour proliferation^10^ and in blockade of fibrosis^11^,^12^. In cardiac diseases, HDACi inhibit arrhythmia^13^ and myocardial infarction^14^,^15^. Many research groups, including ours^16^,^17^, have shown that HDACi also prevent cardiac hypertrophy^17^,^18^. Thus, we had first postulated that HDACi would have preventive effects in VC. On the contrary, however, we found that HDAC inhibition resulted in the enhancement of VC. We therefore further investigated the functional roles of HDACs in the development of VC. Here we report that proteasome-dependent degradation of HDAC1 results in the enhancement of VC, and that MDM2 acts as an E3 ubiquitin ligase of HDAC1 in response to calcification stimuli.

## Results

### Class I HDAC inhibition enhances VC

**Figure 1:**
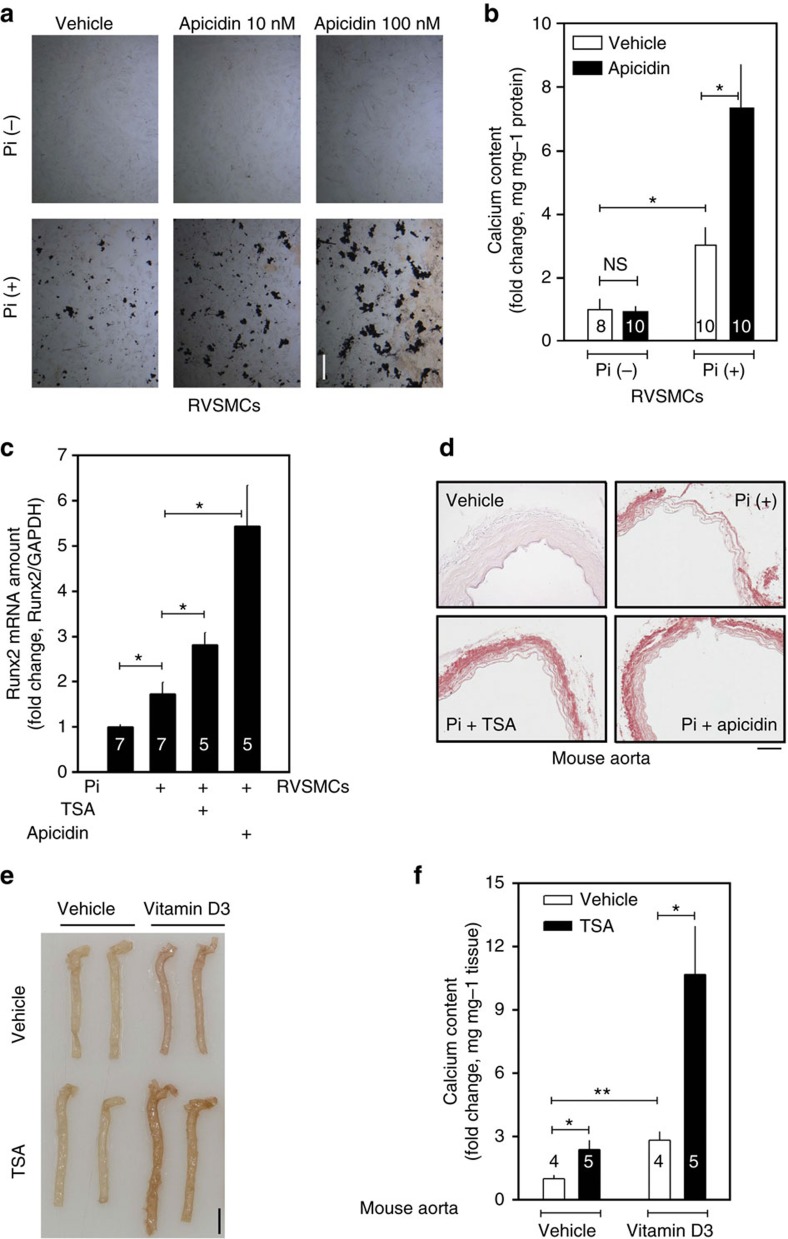
HDAC inhibitors potentiate vascular calcification (VC). (**a**) Apicidin, a class-I-selective HDACi, potentiated the Pi-induced VC in a dose-dependent manner. VC was induced either by inorganic phosphate (Pi) in rat vascular smooth muscle cells (RVSMCs) or by vitamin D_3_ (VD_3_) in mouse. Scale bar, 100 μm. (**b**) Quantification results. Samples (8–10)were measured from three independent experimental sets. (**c**) Both 10 nM TSA and 50 nM apicidin potentiated Pi-induced induction of *Runx2*. Quantitative real-time RT–PCR was performed. Each sample was measured in duplicate and counted as one case (*n*=5–7 from two sets). (**d**) Induction of calcification of aorta *ex vivo* revealed enhancement of VC by Pi. Alizarin red S staining. Scale bar, 100 μm. (**e**) TSA (0.6 mg kg^−1^, intraperitoneally for 9 days) potentiated VC induced by VD_3_ (5 × 10^5^ IU kg^−1^ per day, subcutaneous administered for the first 3 days). Calcification was determined with Alizarin red S staining. Scale bar, 3 mm. (**f**) Quantification results of calcium content in the proximal aorta. Calcium contents from four to five mice in one experimental set were measured. Error bars represent s.e.m. *P<0.05, **P<0.01, Numerals in bar graphs are the numbers of samples.

Treatment of primary cultured rat vascular smooth muscle cells (RVSMCs) with trichostatin A (TSA), a non-class-selective HDACi, did not affect calcification. However, it significantly enhanced inorganic phosphate (Pi, 2 mM)-induced calcium deposition in RVSMCs (Supplementary Fig. 1a), which was quantified with calcium content measurement (Supplementary Fig. 1b). We further studied whether this enhancement of calcium deposition would be repeated with the class-I-selective HDACi apicidin^18^. Although apicidin itself did not induce VC, it potentiated Pi-induced calcium deposition (Fig. 1a,b). Non-class-selective HDACi-mediated enhancement of VC was also recently reported in a cellular model, and an increase in osteogenic Runx2 was elucidated as a possible mechanism^19^. Indeed, in our experimental models, we observed that apicidin, as well as TSA enhanced the expression of *Runx2* (Fig. 1c), which had been known to play a key role in the development of VC^20^. HDACi (10 nM TSA and 50 nM apicidin) and Pi (2 mM) did not affect RVSMC survival as determined by 3-(4,5-dimethylthiazol-2-yl)-2,5-diphenyltetrazolium bromide (MTT) assay (Supplementary Fig. 1c). In an *ex vivo* experimental model in which whole isolated aorta was cultured in DMEM, Pi induced calcium deposition in the aorta, which was then significantly enhanced by either TSA or apicidin (Fig. 1d). We next sought to determine whether inhibition of HDAC potentiates VC in mice. Vitamin D_3_ (VD_3_) injection caused hypercalcemia (Supplementary Fig. 1d). VD_3_-induced VC was enhanced by TSA (Fig. 1e), although the serum calcium level was not changed (Supplementary Fig. 1d). Quantification of calcium content clearly showed that administration of TSA enhanced VD_3_-induced VC (Fig. 1f).

### Loss of HDAC1 potentiates VC

**Figure 2:**
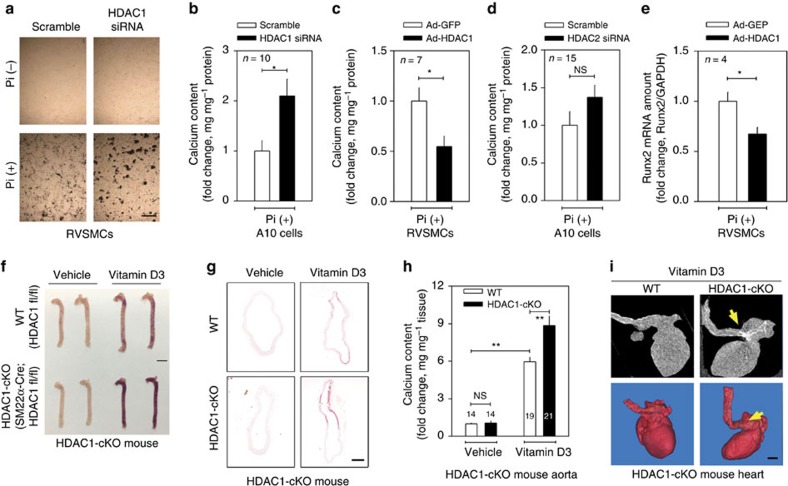
Loss of HDAC1 enhances vascular calcification (VC). (**a**) Transfection of *HDAC1* small interfering RNA (siRNA, 25 nM) potentiated Pi-induced VC. Von Kossa staining. Scale bar, 100 μm. (**b**) Quantification results of calcium content in *HDAC1* siRNA-transfected A10 cells. Ten samples from three independent sets were measured. (**c**) Infection of adenoviral HDAC1 (Ad-HDAC1) to RVSMCs blunted Pi-induced VC. RVSMCs were treated with Ad-HDAC1 (50 MOI), kept in serum-free condition for 24 h and then switched to Pi-containing media. Cells were then treated with Ad-HDAC1 every 2 days (*n*=7 from two sets). (**d**) Reduction of HDAC2 by *HDAC2* siRNA (25 nM) did not potentiate Pi-induced VC (*n*=15 samples from three sets). (**e**) Ad-HDAC1 reduced *Runx2* mRNA amount. Each sample was measured in duplicate and counted as one case (*n*=4 from two sets). (**f**) Vascular smooth muscle cell-specific genetic ablation of *HDAC1* (*SM22α-cre*;*HDAC1*^*fl/fl*^ mice, *HDAC*1-cKO) caused exaggeration of VC induced by administration of VD_3_ in mice, compared with *HDAC1*^*fl/fl*^ control. VD_3_ was administered to 6–8-week-old *HDAC1*-cKO or *HDAC1*^*fl/fl*^ male mice. Alizarin red S staining. Scale bar, 3 mm. (**g**) Horizontal sections of aorta showing VC in *HDAC1*-cKO mice. Scale bar, 200 μm. (**h**) Quantification results of calcium deposition in *HDAC1*-cKO mouse aorta. (**i**) Computed tomography (CT) images showing enhanced calcification in the arch of aorta. Arrows indicate the calcification foci at the proximal aorta and its branches. Upper panels: CT images. Lower panels: three-dimensional reconstruction images. Scale bar, 1 mm. Error bars represent s.e.m. *P<0.05, **P<0.01, NS, not significant. Numerals in bar graphs are the numbers of samples.

Next we questioned which HDAC among the class I HDACs (HDAC1, 2, 3 and 8) is responsible for the potentiation of VC. *HDAC1* short interfering RNA (siRNA) reiterated the effect of the HDACi (Fig. 2a,b; Supplementary Fig. 1e). Adenoviral overexpression of HDAC1 (Ad-HDAC1) caused a decrease in calcium content (Fig. 2c; Supplementary Fig. 1f), but *HDAC2* siRNA did not affect VC (Fig. 2d; Supplementary Fig. 1g). In contrast, forced expression of *HDAC1* with Ad-HDAC1 reduced the expression of *Runx2* (Fig. 2e). The involvement of HDAC1 was further confirmed in *HDAC1* KO mice. Because conventional genetic disruption of *HDAC1* in the whole body results in embryonic lethality^21^, we generated vascular smooth muscle-specific KO of *HDAC1* (*HDAC1*-cKO; Supplementary Fig. 1h). VD_3_-induced calcification was enhanced in *HDAC1*-cKO mice (Fig. 2f). A transverse section of the aorta showed that the calcification area was expanded in VD_3_-treated *HDAC1*-cKO mice (Fig. 2g), which resulted in the enhancement of calcium deposition in the proximal aorta (Fig. 2h). VC was prominent in the proximal aorta and its branches when observed by three-dimensional reconstruction after computed tomography (Fig. 2i, arrowheads). Serum calcium level was not altered in *HDAC1*-cKO (Supplementary Fig. 1i).

### HDAC1 protein amounts are reduced in calcification condition

**Figure 3:**
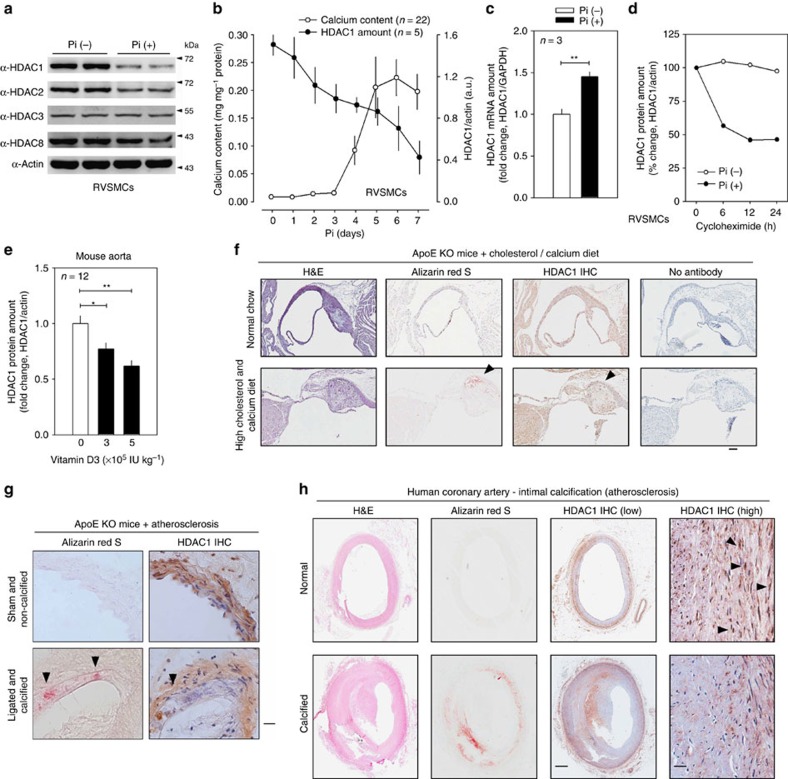
HDAC1 protein, but not mRNA, is reduced in VC. (**a**) Among the class I HDACs, HDAC1 and HDAC2 protein amounts were reduced by Pi treatment. (**b**) Time course of calcium deposition (open circle) and HDAC1 protein reduction (black circle). Note that HDAC1 protein reduction precedes substantial increase in calcium content. Calcium contents were measured in 22 samples from six sets and quantification results of HDAC1 protein amounts were obtained from five samples out of three independent sets of experiments. (**c**) Changes in *HDAC1* mRNA levels. mRNA content was determined with quantitative real-time RT–PCR. Each sample was measured in duplicate and counted as one case (*n*=3 from one set). (**d**) Cycloheximide (CHX) chase study elicited enhancement of HDAC1 protein decay by Pi. After treatment with Pi (2 mM, 6 days), 20 μg ml^−1^ CHX was treated for the indicated interval. Values were averaged from two experiments. (**e**) VD_3_ significantly reduced the protein amount of HDAC1 in the aorta in a dose-dependent manner. Quantification results from 12 western blots from four independent experimental sets. (**f**) Immunohistochemical analysis showed the reduction of HDAC1 in atherosclerosis-associated calcification mouse models. Ten-week-old *ApoE* knockout (KO) male mice were fed a high-cholesterol diet for 10 weeks followed by a high-cholesterol plus calcium diet for the next 7 weeks. The HDAC1 expression level was downregulated in the tissues adjacent to the calcified focus (arrowheads). Scale bar, 100 μm. (**g**) Immunohistochemical analysis showed a reduction of HDAC1 in an alternative atherosclerosis animal model. *ApoE* KO male mouse aorta was subjected to carotid artery ligation to induce sheer stress and atherosclerosis developed. Some mice showed calcified foci (arrowheads) adjacent to the atherosclerotic plaque where HDAC1 expression was lowered. Scale bar, 25 μm. (**h**) HDAC1 protein level was downregulated in atherosclerosis-associated human coronary artery. Arrowheads indicate HDAC1-positive nuclei. Scale bar, 500 μm (low power); 25 μm (high power). *P<0.05, **P<0.01.

Among class I HDACs, HDAC1 and HDAC2 were significantly reduced by Pi in RVSMCs (Fig. 3a). The reductions of HDAC1 and HDAC2 protein amounts were quantified (Supplementary Fig. 2a,b) and the reduction of HDAC1 was greater than that of HDAC2. By time-course analysis, we found that the reduction of HDAC1 (Supplementary Fig. 2c) preceded the substantial increase in calcium deposition that was peaked 5–6 days after Pi treatment (Fig. 3b), which suggests that reduction of HDAC1 is a cause of VC. We also observed that Pi-induced calcium deposition (Supplementary Fig. 2d) causes the reduction of protein amount of HDAC1 (Supplementary Fig. 2e) in human coronary artery smooth muscle cells (HCASMCs).

In other *in vitro* calcification models, osteogenic medium^22^ (OM) induced VC (Supplementary Fig. 2f), which also caused a reduction of HDAC1 (Supplementary Fig. 2g). CaCl_2_ (8 mM), but not β-glycerophosphate (10 mM), significantly reduced HDAC1 amounts (Supplementary Fig. 2h). Phosphonoformic acid, a Pi transporter inhibitor^23^, completely blocked VC (Supplementary Fig. 2i), which resulted in recovery of the reduced HDAC1 protein amounts (Supplementary Fig. 2j). Next, we questioned whether the decrease in the protein amounts resulted from a decrease in transcription of HDAC1 or an increase in protein degradation. Interestingly, transcript levels of all class I HDACs were increased by Pi, whereas class II HDACs were not altered (Fig. 3c; Supplementary Fig. 2k). When *de novo* synthesis of protein was blocked by cycloheximide, HDAC1 decay was significantly accelerated (Fig. 3d).

In VD_3_-treated mice, the protein amounts of HDAC1 were significantly reduced in aorta in a dose-dependent manner (Fig. 3e). The reduction of HDAC1 was confirmed by histologic evaluation (Supplementary Fig. 3a), and was further quantified (Supplementary Fig. 3b). In contrast, we were not able to observe any significant reduction of messenger RNA (mRNA) levels (Supplementary Fig. 3c,d).

Clinically, atherosclerosis is closely related to the development of intimal VC^24^. Thus, we examined whether HDAC1 expression was also reduced in the atherosclerosis-associated VC models. As described previously^25^, a diet high in cholesterol plus calcium markedly induced calcification (Fig. 3f), as well as atherosclerosis in *ApoE* KO mice. In the localized area where the severe calcification developed, HDAC1 expression was decreased (Fig. 3f, arrowheads). VC is often associated with atherosclerosis even without apparent calcification stimuli^26^. Thus, we investigated whether HDAC1 was also reduced in atherosclerosis-associated calcification without administration of VD_3_ or calcium. Carotid artery ligation in *ApoE* KO mice significantly induced atheromatous plaques as described previously^27^. Some of the mice (∼30%) developed localized calcification (arrowheads in lower left panel in Fig. 3g), regardless of the severity of atheroma, and HDAC1 expression was downregulated (lower right panel in Fig. 3g) in the ligated mice compared with the non-ligation group (upper right panel). Quantification of the histological analysis is provided in Supplementary Fig. 3e. For further evaluation in a human disease, calcified human coronary artery samples were used for HDAC1 immunohistochemistry. Compared with the expression in coronary artery from normal patients, severe calcification reduced the expression of HDAC1 (Fig. 3h). The changes in the expression of HDAC1 are quantified in Supplementary Fig. 3f. The mRNA level of *HDAC1*, however, was not altered in calcified human coronary artery compared with normal (Supplementary Fig. 3g). These results suggest that reduction of HDAC1 protein, but not mRNA, is associated with VC.

### HDAC1 is degraded through K74 ubiquitination

**Figure 4:**
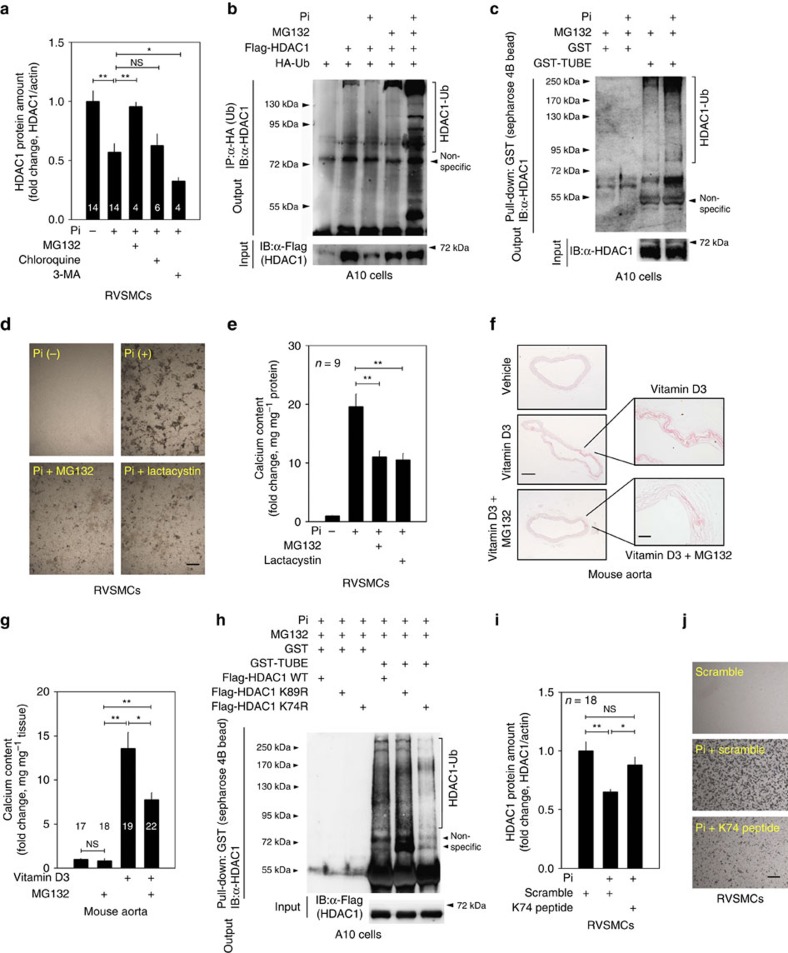
HDAC1 is ubiquitinated in VC. (**a**) MG132 (10 μM, proteasome inhibitor) but not chloroquine (100 μM, lysosome inhibitor) nor 3-methyladenine (2 mM, autophagy inhibitor) blocked Pi-induced reduction of HDAC1. (**b**) Ubiquitination of HDAC1 was enhanced by Pi treatment for 3 days (fourth versus 5th lane). Twenty-four hours after transfection with Flag-HDAC1 and HA-Ub, A10 cells were treated with Pi. MG132 was added 4 h before collecting. The cell lysates were immunoprecipitated with anti-HA antibody and immunoblotted with anti-HDAC1 antibody. Western blots (4–14) from four to eight independent sets were analysed. (**c**) Tandem ubiquitin-binding entities (TUBEs) assay to check K48 ubiquitination of HDAC1 in response to Pi. Pi-treated RVSMCs were subjected to GST pull-down assay with either GST only or GST-TUBE and then HDAC1 was detected with immunoblot. HDAC1-bound multiple ubiquitin conjugation was detected. A10 cells were treated with Pi for 3 days. (**d**) Von Kossa staining showed that Pi-induced RVSMC calcification was blunted by either MG132 (10 μM) or lactacystin (10 μM). Scale bar, 100 μm. (**e**) Quantification results (*n*=9 from three sets). (**f**) Administration of MG132 to mice blunted VD_3_-induced VC. MG132 (2.5 mg kg^−1^ per day, intraperitoneally) was administered for 9 days, whereas VD_3_ was treated for the first 3 days. Scale bar, 250 μm (low power); 50 μm (high power). (**g**) Quantification of calcium deposition in proximal aorta (17–22 aortic samples from four to six independent experimental sets). (**h**) TUBE assay revealed that HDAC1 ubiquitination is dependent on K74. Treatment of A10 cells with Pi for 3 days failed to induce ubiquitination of HDAC1 K74R, whereas it successfully induced ubiquitination of HDAC1 K89R. (**i**) Decoy peptide spanning HDAC1 K74 attenuated Pi-induced reduction of HDAC1 protein amount (eight samples from two sets). (**j**) K74 peptide attenuated Pi-induced calcium deposition. Von Kossa staining was performed. Scale bar, 100 μm. *P<0.05, **P<0.01, NS. not significant. Numerals in bar graphs are the numbers of samples.

We next studied the mechanism of the degradation of HDAC1 in VC. As shown in Fig. 4a, Pi-induced reduction of HDAC1 was markedly attenuated by treatment with MG132, a proteasome inhibitor, but not by treatment with inhibitors of lysosome (chloroquine) or autophagy (3-methyladenine). Alternative proteasome inhibitors also prevented HDAC1 degradation (Supplementary Fig. 4a). By the use of immunoprecipitation-based ubiquitination assays, we found that in the presence of MG132 and ubiquitin, Flag-tagged exogenous HDAC1 was ubiquitinated in A10 cells, a RVSMC line (4th lane in Fig. 4b, immunoprecipitation with ubiquitin (anti-HA antibody) and immunoblotting with HDAC1 (anti-HDAC1 antibody)). Interestingly, the ubiquitination of HDAC1 was further enhanced by Pi (5th lane in Fig. 4b).

To further rule out non-proteolytic ubiquitination of HDAC1, a tandem ubiquitin-binding entities (TUBEs)^28^,^29^ assay was performed. The TUBE assay recognizes four sequential conjugations of ubiquitin molecules (tetra-ubiquitin), that is, ubiquitination. With Pi-treated A10 cells, a pull-down assay was performed with either glutathione *S*-transferase (GST) or GST-TUBE, and then the precipitates were subjected to immunoblotting with anti-HDAC1 antibody. The TUBE assay showed that HDAC1 ubiquitination is increased by Pi treatment in A10 cells (Fig. 4c). When RVSMCs were treated with MG132 and lactacystin, an alternative proteasome inhibitor, the amount of calcium deposition induced by Pi was halved (Fig. 4d,e). The effect of proteasome inhibitor was also investigated *in vivo*; intraperitoneal administration of MG132 significantly reduced VD_3_-induced calcium deposition (Fig. 4f,g) and prevented the reduction of HDAC1 expression (Supplementary Fig. 4b), whereas it did not affect the serum calcium level (Supplementary Fig. 4c).

Next, we isolated the lysine residues responsible for the HDAC1 ubiquitination. Using bioinformatics analysis ( www.phosphosite.org), we investigated K74 and K89, which are well conserved (Supplementary Fig. 4d). We generated two mutant mammalian expression vectors of HDAC1 (K74R and K89R) and performed GST pull-down-based TUBE assay in A10 cells transfected with either *HDAC1* wild-type or *HDAC1* mutants in the presence of MG132 and Pi stimulation. The results showed that HDAC1 ubiquitination was blunted in HDAC1 K74R, whereas HDAC1 K89R ubiquitination was not attenuated (Fig. 4h). Indeed, in the absence of MG132, Pi-induced reduction of HDAC1 protein was blunted in HDAC1 K74R-transfected A10 cells (Supplementary Fig. 4e).

We further questioned whether specific blockade of HDAC1 ubiquitination might prevent VC. We designed a 15 amino-acid-long synthetic peptide spanning the K74 residue. This peptide was linked with fluorescein isothiocyanate-conjugated nuclear localization signal (NLS) peptide^30^ (Supplementary Fig. 4f). Compared with scramble peptide conjugated with fluorescein isothiocyanate, the synthetic peptide successfully incorporated in the nucleus in RVSMCs (Supplementary Fig. 4g). K74 peptide successfully blocked the Pi-induced reduction of the HDAC1 protein amount (Fig. 4i) and attenuated calcium deposition in RVSMCs (Fig. 4j). These results suggested that the synthetic peptide spanning K74 worked as a decoy peptide to prevent VC in RVSMCs.

### MDM2 induces ubiquitination of HDAC1 in VSMCs

**Figure 5:**
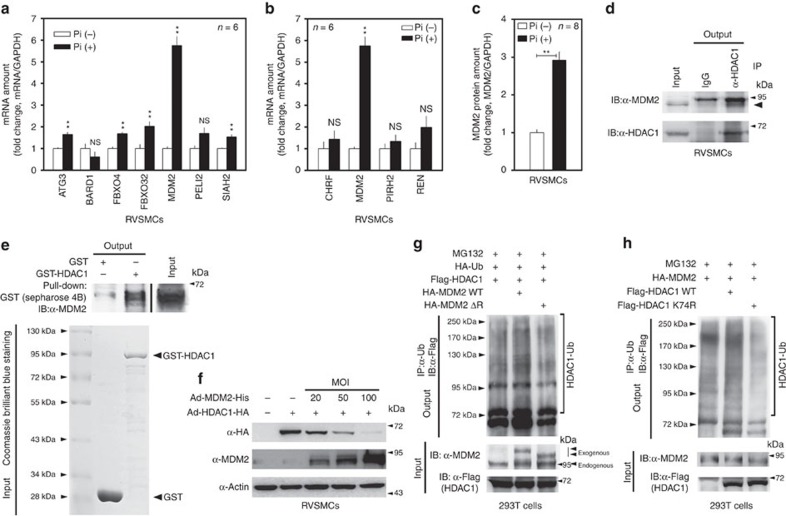
MDM2 E3 ligase induces degradation of HDAC1. (**a**) Pi-induced changes in the mRNA level of seven candidate genes according to cDNA microarray analysis (*n*=6 from two sets). Each sample was measured in duplicate. (**b**) Pi-induced changes in the mRNA level of four candidate genes on the basis of reports in the literature of HDAC1-specific E3 ligases (*n*=6 from two sets). Note that MDM2 was listed among both the cDNA microarray-based candidates (**a**) and the literature-based ones (**b**). (**c**) Pi significantly increased MDM2 protein expression (*n*=8 from two sets). (**d**) Immunoprecipitation analysis showed that endogenous HDAC1 physically associated with endogenous MDM2 in RVSMCs. (**e**) GST pull-down assay to show the direct interaction between MDM2 and HDAC1. Both GST-HDAC1 and His-MDM2 proteins were generated from *E. coli* and then utilized for GST pull-down assay. MDM2-His was recruited by Sepharose 4B-bound GST-HDAC1. (**f**) Adenoviral infection of MDM2 induced dose-dependent reduction of HDAC1 protein in RVSMCs. Cells were treated with an equal amount of Ad-HDAC1 (20 MOI) in each case. (**g**) Transfection of wild-type MDM2 to 293T cells enhanced the ubiquitination of HDAC1 (second lane). However, transfection of MDM2ΔR that lacked the RING domain for E3 ligase activity failed to do so (third lane). Flag-HDAC1 and HA-Ub with either HA-MDM2 or HA-MDM2ΔR were transfected and maintained for 2 days. Cells were treated with MG132 4 h before collecting. The cell lysates were immunoprecipitated with Ub and immunoblotted with HDAC1. (**h**) MDM2-induced HDAC1 ubiquitination was attenuated in HDAC1 K74R. Ub assay was performed. **P<0.01, NS, not significant.

E3 ligases often determine the specificity of ubiquitin proteasome protein degradation. To find the specific E3 ligase for HDAC1 degradation in VC, we postulated that the E3 ligase might (1) be upregulated in VC, (2) induce calcification and (3) ubiquitinate HDAC1 in RVSMCs. We first performed complementary DNA (cDNA) microarray with Pi-treated RVSMCs (Supplementary Fig. 5a,b). Seven E3 ligases were included in the dysregulated genes. Among those E3 ligases, two F-box proteins (*FBXO4* and *FBXO32*) and *MDM2* were upregulated by Pi (Supplementary Fig. 5c). We further confirmed the changes in amounts of those 7 E3 ligases by quantitative PCR with reverse transcription (qRT–PCR) and found that *MDM2* was most upregulated (Fig. 5a). We checked the transcript levels of four different E3 ligases previously reported in the literature to ubiquitinate HDAC1: *CHFR*^31^, *MDM2* (ref. 32), *PIRH2* (ref. 33) and *REN*^34^. Only *MDM2* was significantly increased by Pi treatment in RVSMCs (Fig. 5b). Pi treatment for 3 or 6 days also increased the protein amount of MDM2 in RVSMCs (Fig. 5c; Supplementary Fig. 6a). We measured changes in the protein amounts of the other four E3 ligases (ATG3, FBXO4, FBXO32 and SIAH2), the mRNA amounts of which were significantly increased among cDNA microarray-based candidates; none of the other E3 ligases were significantly altered at the protein level (Supplementary Fig. 6b).

Next, we checked whether HDAC1 and MDM2 could physically interact. The immunoprecipitation assay showed that exogenous HDAC1 physically interacted with exogenous MDM2 in 293T cells (Supplementary Fig. 6c,d). The association of both proteins was further observed with endogenous proteins in RVSMCs (Fig. 5d). To confirm the direct interaction between MDM2 and HDAC1, we utilized the GST pull-down assay in the cell-free condition. Bacterially overexpressed His-MDM2 protein was successfully pulled down by Sepharose 4B-bound GST-HDAC1 (second lane in the upper gel in Fig. 5e), which suggests that no other protein is involved in the interaction. Infection of adeno-MDM2 (Ad-MDM2) reduced exogenous HDAC1 protein expression by Ad-HDAC1 in a dose-dependent manner in RVSMCs (Fig. 5f). Transient transfection of HA-MDM2 also potentiated Pi-induced calcium deposition in A10 cells (Supplementary Fig. 6e).

MDM2 possesses a RING domain that is indispensable for ubiquitination activity^35^. Deletion of the RING domain of MDM2 (MDM2ΔR) results in the loss of E3 ligase activity^36^. Compared with wild-type MDM2, which induced ubiquitination of HDAC1, MDM2ΔR failed to do so (Fig. 5g). MDM2 failed to induce ubiquitination of HDAC1 K74R (Fig. 5h), but did induce ubiquitination of K89 (Supplementary Fig. 6f). These results suggest that Pi induces MDM2 and the increase in MDM2 results in the ubiquitination of HDAC1 K74 in RVSMCs.

### MDM2 induces calcium deposition in a p53-independent manner

**Figure 6:**
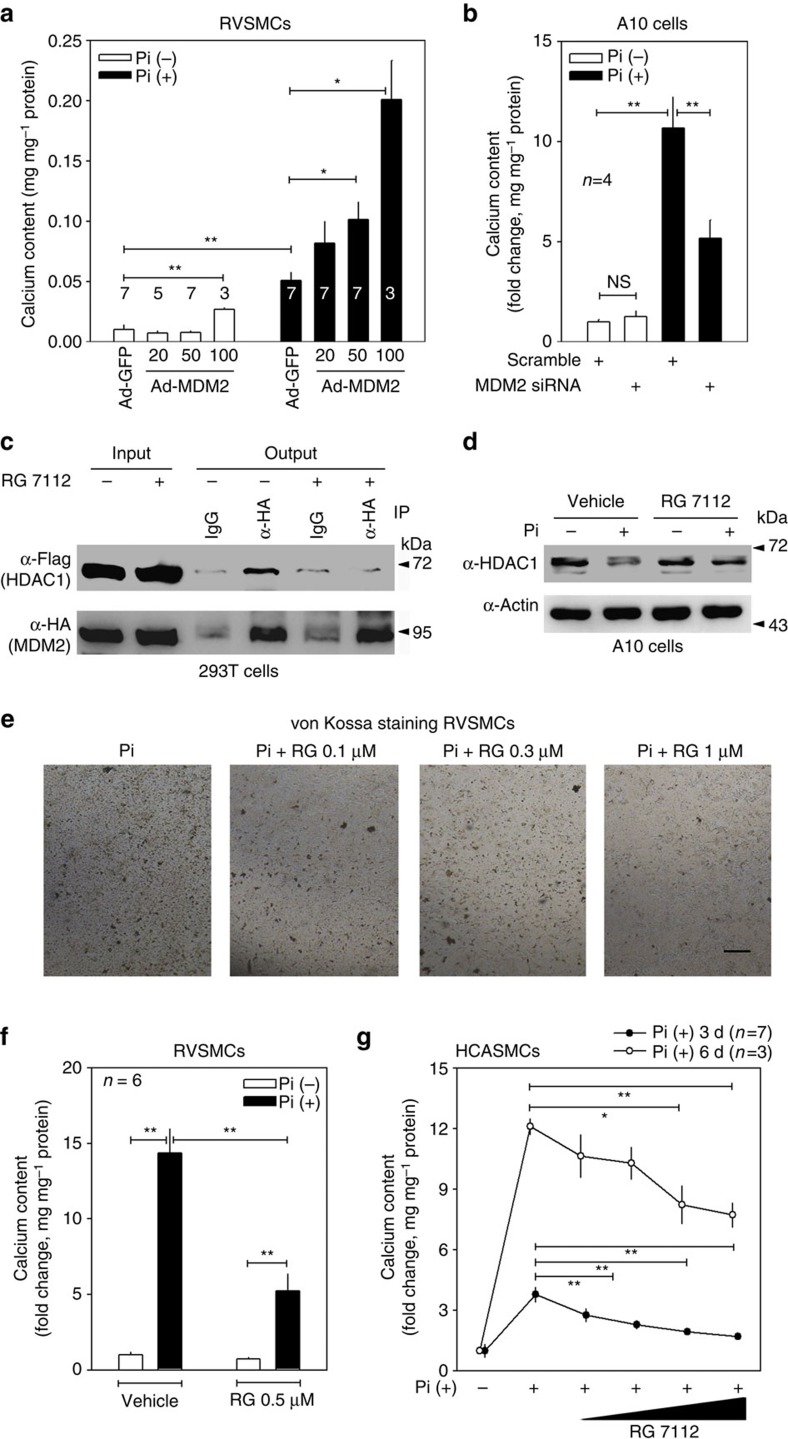
MDM2 induces VC. (**a**) Adenoviral infection of MDM2 enhanced Pi-induced VC in a dose-dependent manner. Numbers under the horizontal axis are the MOI of adeno-MDM2 (*n*=3–7 from one to two experimental sets). (**b**) *MDM2* siRNA blunted Pi-induced VC in A10 cells (*n*=9 from three sets). Either *MDM2* siRNA or scramble was transfected with Lipofectamine RNAiMAX. (**c**) Immunoprecipitation analysis to show that RG 7112 (RG), an MDM2 inhibitor, interfered with the association of HDAC1 with MDM2. Note the physical interaction between HDAC1 and MDM2 (fourth lane) was attenuated by RG treatment (sixth lane). HA-MDM2 and Flag-HDAC1 were transfected and either RG (2.5 μM) or vehicle was treated for 24 h in 293T cells. (**d**) RG (0.1 μM) blocked the Pi-induced reduction of HDAC1 protein amount in A10 cells. (**e**) RG attenuated Pi-induced VC in RVSMCs in a dose-dependent manner. Pi-containing media with either RG or vehicle were replaced every 2 days for 6 days and von Kossa staining was performed. Scale bar, 100 μm. (**f**) Quantification results to show the inhibitory effect of RG on Pi-induced VC. RG (0.5 μM) significantly reduced the calcium deposition in RVSMCs (*n*=6 from two sets). (**g**) Dose-dependent attenuation of calcium deposition by RG compound (0.1–3 μM) in human coronary artery smooth muscle cells (HCASMCs). Pi was treated for 3 days (filled circle, *n*=7 from two sets) or 6 days (open circle, *n*=3 from one set). *P<0.05, **P<0.01.

Next, we questioned whether MDM2 can induce VC. Infection of Ad-MDM2 induced calcium deposition in RVSMCs only at a higher dose (fourth column in Fig. 6a). However, under Pi treatment, Ad-MDM2 significantly potentiated the calcium deposition in a dose-dependent manner in RVSMCs (fifth to eighth columns in Fig. 6a). In addition, *MDM2* siRNA (Supplementary Fig. 7a) significantly attenuated Pi-induced calcium deposition in A10 cells (Fig. 6b).

[(4S,5R)-2-(4-tert-butyl-2-ethoxyphenyl)-4,5-bis(4-chlorophenyl)-4,5-dimethylimidazol-1-yl]-[4-(3-methylsulfonylpropyl)piperazin-1-yl]methanone (RG 7112, (RG)), a member of the Nutlin family, has been recently developed as a novel inhibitor of MDM2. RG stabilizes p53 to elicit anticancer activity^37^. As a mechanism, it has been reported that RG interferes with the association of MDM2 with p53 by masking the binding surface of MDM2 (ref. 38). Thus, we checked whether RG could also block MDM2 activity by interfering with binding to HDAC1 and thereby whether RG could prevent VC. Treatment with RG (2.5 μM) resulted in the dissociation of HDAC1 and MDM2 (Fig. 6c). RG (0.1 μM) prevented the Pi-induced reduction of HDAC1 in A10 cells (Fig. 6d). Von Kossa staining revealed that RG blocked calcium deposition in a dose-dependent manner in RVSMCs (Fig. 6e). RG (0.5 μM) blocked VC in RVSMCs (Fig. 6f). In HCASMCs, calcium deposition by Pi treatment for either 3 or 6 days was significantly attenuated in RG in a dose-dependent manner (Fig. 6g).

The molecular mechanism of VC is somewhat similar to that of osteogenic differentiation. The role of MDM2 in osteoblast differentiation has been reported; bone formation fails in mice lacking MDM2 in osteoblast progenitor cells but can be successfully rescued by p53 inactivation^39^. We investigated the possible involvement of MDM2-mediated regulation of p53 in VC. VD_3_ administration reduced p53 protein amounts in mouse aorta (Supplementary Fig. 7b). In RVSMCs, Pi also reduced p53; however, interestingly, MDM2 siRNA failed to block the Pi-induced reduction of p53 (Supplementary Fig. 7c). We further checked whether reduction of p53 affects VC. Reduction of p53 with siRNA (Supplementary Fig. 7d) did not alter the Pi-induced VC (Supplementary Fig. 7e). Likewise, pifithrin-α, a p53 inhibitor^40^, did not affect the VC (Supplementary Fig. 7f). These results suggest that MDM2 is not involved in Pi-induced p53 reduction, and that the reduction of p53 does not participate in the VC at least in our experimental model.

### MDM2 causes VC *in vivo* models

**Figure 7:**
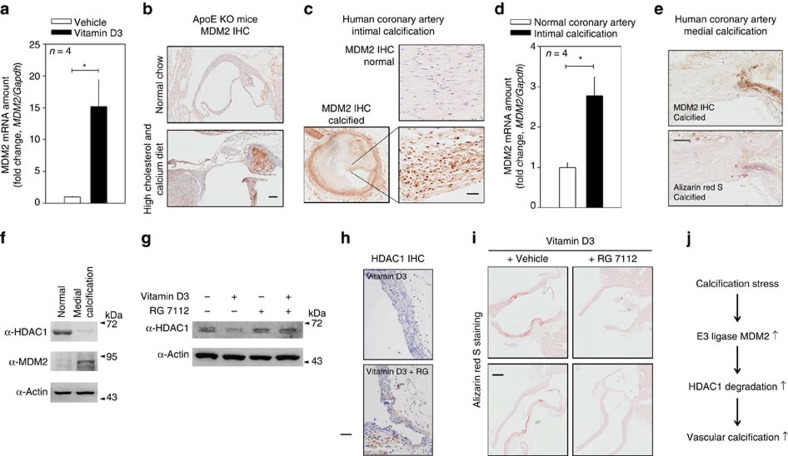
MDM2 is upregulated in VC models and inhibition of MDM2 activity reduces VC in mice. (**a**) *MDM2* mRNA level was upregulated in the aorta of VD_3_-administered mice (four mice from two sets). Each sample was measured in duplicate. (**b**) Immunohistochemical analysis showing MDM2 expression in *ApoE* mice fed high cholesterol and calcium as explained in Fig. 3f. Scale bar, 100 μm. (**c**) Immunohistochemical analysis showing that MDM2 expression is increased in the atherosclerosis-associated intimal calcification model of human coronary artery. The adjacent section slide was used with Fig. 3h. Scale bar, 50 μm. (**d**) Quantitative real-time RT–PCR results show that *MDM2* mRNA level is significantly increased in the intimal calcification model of human coronary artery (four samples in duplicate). (**e**) MDM2 expression was increased at the site of calcification in human coronary artery with medial calcification in association with diabetes. Scale bar, 200 μm. (f) HDAC1 was downregulated, whereas MDM2 was upregulated, in human coronary artery sample with medial calcification. (**g**) Administration of RG (intraperitoneally, 50 mg kg^−1^ per day, 9 days) prevented VD_3_-induced reduction of HDAC1 protein amount in mouse aorta. (**h**) Immunohistochemical analysis showing HDAC1 expression in VD_3_-treated mice. Note that nuclear expression of HDAC1 was restored by administration of RG. Scale bar, 50 μm. (**i**) Intraperitoneal administration of RG prevented VD_3_-induced VC in the ascending aorta. Scale bar, 250 μm. (**j**) Diagram of MDM2/HDAC1 signal cascade in VC. *P<0.05.

We next studied the function of MDM2 *in vivo* models. In VD_3_-administered mouse aorta, the transcript level of *MDM2* was significantly increased (Fig. 7a). Histological analysis with quantification also showed that MDM2 was increased in the aorta of VD_3_-administered mice (Supplementary Fig. 8a,b). MDM2 expression was also increased in both *ApoE* KO mice fed a diet high in cholesterol and calcium (Fig. 7b) and *ApoE* KO carotid artery ligation mice (Supplementary Fig. 8c,d). The increases in MDM2 expression were further examined in two human calcification models of intimal and medial calcification. Histological analysis showed that MDM2 expression was increased in the intimal calcification model (Fig. 7c; Supplementary Fig. 8e). The increase in *MDM2* mRNA level was also observed (Fig. 7d). Because metabolic diseases such as diabetes mellitus or chronic renal failure might cause medial calcification^23^,^26^, we investigated whether the increase in MDM2 is also associated with medial calcification in human coronary artery. We observed significant MDM2-positive signals near the calcification site (Fig. 7e). Indeed, MDM2 protein expression was increased, whereas HDAC1 was downregulated in calcified human coronary artery (Fig. 7f).

Next, we examined whether blockade of MDM2 activity by RG compound could result in the prevention of VC in VD_3_-administered mice model. Intraperitoneal administration of RG to mice restored the VD_3_-induced reduction of HDAC1 in the arch of aorta as determined with immunoblot (Fig. 7g). Immunohistochemistry analysis for HDAC1 also showed that HDAC1 expression was restored by administration of RG (Fig. 7h). Most importantly, intraperitoneal administration of RG blocked the VD_3_-induced VC in mouse, as determined by Alizarin red S staining (Fig. 7i). These results suggest that chemical inhibition of MDM2 may prevent VC.

## Discussion

Here we elucidate the previously unknown MDM2/HDAC1 pathway in the development of VC (Fig. 7j). In this pathway, under conditions that lead to calcification, the expression of MDM2 is induced, and MDM2 acts as an E3 ligase to ubiquitinate HDAC1. The reduction of HDAC1 contributes to the potentiation of VC, which is somewhat coincident with our result that HDACi exaggerates VC. These findings show not only an HDAC-related regulation mechanism but also novel MDM2-mediated proteasomal degradation in VC. This observation provides clues for potential therapeutic application of the MDM2/HDAC1 signal cascade, either by utilizing decoy peptide against HDAC1 degradation or by MDM2 inhibitor.

These findings are of major significance. (1) In this report, we postulate that HDACi might have potential adverse effects of VC. (2) We first demonstrated that ubiquitination mediates VC; blockade of proteasomal degradation by diverse inhibitors of ubiquitination attenuated VC *in vitro* and *in vivo* models. (3) We show that MDM2/HDAC1 may represent an attractive target for drug development against VC by demonstrating the role of MDM2/HDAC1 signalling cascade in the cardiovascular pathophysiology. Indeed, we demonstrated that blockades of either HDAC1 degradation or MDM2 activity can attenuate VC. Since MDM2 inhibitors such as nutlin-3 or RG 7112 are now being under extensive investigation for the treatment of cancer by recent research works, application of those drugs for the treatment of VC may be anticipated.

To date, only one report has shown the possible involvement of ubiquitination in VC; Nedd4 E3 ubiquitin ligase was shown to negatively regulate Pi-induced VC by ubiquitination of Smad1 in cellular models *in vitro*^41^. That report contradicts our present work concerning whether ubiquitination may enhance VC; those authors observed the development of VC by specific inhibition of Nedd4. Utilizing *in vivo* and *in vitro* models in the present study, however, we found that overall inhibition of ubiquitination either in RVSMCs or in mice prevents VC, which is mediated at least in part by the MDM2-mediated degradation of HDAC1.

MDM2 is known to induce ubiquitination of several target proteins, with the most well-known target of MDM2 E3 ligase being p53 (refs 36, 42). Upregulation of MDM2 and repression of p53 activity is highly associated with cancer development^43^. Indeed, MDM2 is clinically utilized as a marker of certain types of soft tissue cancer such as liposarcoma^44^. Thus, MDM2 inhibitors such as RG or nutlin-3 are in phase I clinical trials^38^. In bone-selective MDM2 KO mice, the improperly increased p53 activity causes an increase in p21 expression, which results in osteogenic failure in embryo. Thus, during the bone development, MDM2-mediated inhibition of p53 activity and the following decrease in p21 transcription are indispensable for the proper expression of Runx2 in osteoblasts and skeletal development^39^. In contrast, however, in the present study, we observed that p53 was downregulated by triggers of calcification in an MDM2-indepenent manner, and that the reduction of p53 activity did not cause VC. Thus, in contrast with the p53-dependent pathway in bone formation in embryo, our results strongly suggest that an increase in MDM2 and subsequent reduction of HDAC1 contributes to VC.

It should be noted that the VC models in our experiments, such as VC induced by Pi treatment or high-dose vitamin D3 administration, may not be clinically relevant to human VC. Likewise, the mechanism shown by these models may differ in human VC. The simple phenotypic coincidence of downregulation of HDAC1 and upregulation of MDM2 in human coronary artery VC samples may not implicate this MDM2/HDAC1 pathway in human VC. Further studies using a large number of human VC models or more clinically relevant animal models are needed to adequately address this issue. Nonetheless, our report does suggest a plausible mechanism of MDM2/HDAC1 in VC.

As transcriptional regulators, HDACi are potential therapeutics in cancer and neurological disorders. HDACi also promote osteoblastic maturation^45^. Among the HDACs in osteogenesis process, reduction of HDAC1 in osteoblast induces bone formation by the transcriptional activation of Runx2 (ref. 46), a master regulator of osteogenesis^47^. Although it has not been clearly demonstrated which HDAC is involved, HDACi induce calcification in VSMCs by inducing Runx2 and alkaline phosphatase^19^. In the present study, reduction of HDAC1 and inhibition of HDAC activity induced ectopic calcification in the vasculature. It should be noted that HDACi are being currently used in anticancer therapeutics. In addition, possible clinical applications of HDACi in diverse heart diseases, such as cardiac hypertrophy^16^,^17^,^48^, fibrosis^11^,^12^, arrhythmia^13^ and myocardial infarction^14^,^15^, and neurologic diseases such as epilepsy^49^, are being highlighted by many research groups including ours. Considering our results, however, the likeliness of increase of VC by HDACi should be noted as a potential adverse effect for future drug development.

## Methods

### Reagents

Antibodies against anti-HDAC2 (1:1,000, ab12169), HDAC3 (1:1,000, ab16047), HDAC8 (1:1,000, ab137474), FBXO4 (1:1,000, ab83318) and MDM2 (1:1,000, ab3110) were from Abcam (Abcam, Cambridge, UK); Flag (1:1,000, F7425 and F1804) and actin (1:1,000, A2066) were from Sigma (Sigma-Aldrich, St Louis, MO, USA); HDAC1 (1:1,000, sc7872 and 05–100) was from either Santa Cruz Biotechnology (Santa Cruz Biotechnology, Santa Cruz, CA, USA) or Millipore (EMD Millipore, Billerica, MA, USA); ATG3 (1:1,000, sc-70139), SIAH2 (1:1,000, sc-5507), Ub (1:1,000, sc8017), and Gapdh (1:1,000, sc16574) were from Santa Cruz Biotechnology; Ub (1:1,000, 3936) was from Cell Signaling (Cell Signaling Technology, Denver, MA, USA); FBXO32 (1:1,000, AP2041) was from ECM Biosciences (ECM Biosciences, Versailles, KY, USA); and HA (1:1,000, 11583816001) was from Roche Applied Science (Roche Applied Science, Indianapolis, IL, USA). Anti-mouse (1:5,000, 7076S) or anti-rabbit (1:5,000, 7074S) IgG peroxidase-conjugated secondary antibodies were purchased from Sigma (Sigma-Aldrich).

Cholecalciferol (vitamin D_3_), TSA, apicidin, phosphonoformic acid, pifithrin-α and 2,2,2-tribromoethanol were purchased from Sigma (Sigma-Aldrich). MG132 was purchased from Calbiochem (EMD Millipore). Chloroquine, 3-methyladenine, lactacystin and epoxomicin were purchased from Sigma (Sigma-Aldrich). *N*-[*N*-(*N*-Acetyl-L-leucyl)-L-leucyl]-L-norleucine was purchased from Santa Cruz Biotechnology. *HDAC1* siRNA, *HDAC2* siRNA, *MDM2* siRNA and scramble were purchased from Dharmacon (Thermo Fisher Scientific, Waltham, MA, USA). *p53* siRNA was purchased from Bioneer (Daejeon, Korea). Decoy peptide was synthesized by Peptron (Daejeon, Korea). RG 7112 compound was purchased from ApexBio Technology (Apexbio Technology LLC, Houston, TX, USA).

### Plasmids

*pBJ5.1-Flag-HDAC1* was kindly gifted from Professor Jonathan A. Epstein (University of Pennsylvania, Philadelphia, PA, USA). *pCMV-MDM2* and *pcDNA3-HA-Ub* were kindly provided by Dr Ki Sun Kwon (Korea Research Institute of Bioscience & Biotechnology, Daejeon, Korea). *pcDNA6-3xHA-MDM2-Myc*, *pGEX-HDAC1* and *pET28-MDM2* were generated by PCR-based subcloning (CosmoGeneTech, Seoul, Korea). RING finger domain-deleted *pcDNA6-3xHA-MDM2-Myc* (*MDM2ΔR*), *pBJ5.1-Flag-HDAC1 K74R* and *pBJ5.1-Flag-HDAC1 K89R* were constructed by site-directed mutagenesis (CosmoGeneTech). All plasmids were checked before the use by direct sequencing.

### Cell cultures

RVSMCs were isolated from rat thoracic aorta of 6–7-week-old Sprague-Dawley male rats. The aorta was washed using sterilized ice-cold PBS and incubated in 1 ml 0.2% collagenase I solution in Ham's F12 medium at 37 °C for 30 min. The aortas were opened longitudinally and the intima scraped on luminal surface. Tissue samples were minced into small pieces in dissection medium with Ham's F12 media containing 300 U ml^−1^ penicillin and 300 U ml^−1^ streptomycin, and incubated the dissected tissues in 0.2% collagenase I solution at 37 °C for 30 min with shaking. The dissected tissues were attached to dish and cultured 10% fetal bovine serum (FBS) in DMEM with antibiotics at 37 °C in a humidified atmosphere with 5% CO_2_. RVSMCs were used at passages 2–6. A10 cells were purchased from American Type Culture Collection (CRL-1476, Manassas, VA, USA) and have been used as models of RVSMCs^50^. A10 cells were derived from embryonic rat aorta^51^ and were maintained in 10% FBS in DMEM with antibiotics. Human embryonic kidney 293T cells were obtained from the Seoul Korean Cell Line Bank (21573, Seoul, Korea) and were maintained in 10% FBS in DMEM with antibiotics. HCASMCs were purchased from Gibco (Invitrogen, C-017-5C, Carlsbad, CA, USA) and were grown in Medium 231 with smooth muscle growth supplement.

### Adenoviral GFP, HDAC1 and MDM2

HDAC1 (096643A) and MDM2 (106880A) adenovirus were purchased from Applied Biological Materials (Richmond, BC, Canada). Titre of adenoviruses was determined by the use of the Adeno-X Rapid Titer kit (Clontech Laboratories, Mountain View, CA, USA). The expression of protein was examined by the infection of adenoviral GFP.

### cDNA microarray

To investigate the alteration of the expression of E3 ligase in response to calcification stresses, calcification was induced in RVSMCs treated with inorganic phosphate (Pi) and the mRNA preparation was subjected to cDNA microarray analysis (Rat GE 4 × 44k v3 microarray chip, G2519F, Agilent, Santa Clara, CA, USA). To reduce the variation between sample preparations, two independent sets of experiments were performed and analysed (Genomictree, Daejeon, Korea). Gene expression data have been deposited in the GEO database under accession code GSE74755.

### Induction of VC *in vitro* and *ex vivo*

For the induction of VC in RVSMCs, the cells cultured in growth medium were switched to calcification medium containing 2 mM Pi (pH 7.4) for up to 3 or 6 days. The medium was changed every 2 days. The first day of culture in the calcification medium was defined as day 0 and calcium deposition was determined after the cells were washed twice with 1 × PBS at day 6.

VC was also induced by treatment of RVSMCs with OM. OM was generated by adding 100 nM dexamethasone, 1 μM insulin, 50 μg ml^−1^ ascorbic acid and 10 mM β-glycerophosphate with 8 mM CaCl_2_ to culture media. VC was induced for 3–21 days.

For *ex vivo* experiments, the 6-week-old C57BL/6 male mice were killed under anaesthesia with 2,2,2-tribromoethanol (300 mg kg^−1^, intraperitoneally). The heart and thoracic aortas were dissected down to the renal arteries and removed in 1 × PBS including antibiotics. The heart and aortic root were separated from the more distal aorta^52^. The separated aortas were plated on cell culture plates (60 mm) and cultured in DMEM with 10% FBS and penicillin–streptomycin solution (growth medium). The aortas were then switched to a growth medium containing 2 mM Pi (pH 7.4) for up to 6 days. The medium was changed every 2 days. The first day of culture in the calcification medium was defined as day 0. The aortas were washed twice with 1 × PBS and calcium deposition was determined.

Either K74 decoy peptide or scramble was treated to RVSMCs at the concentration of 100 nM for 6 days. The peptide was replenished every 2 days when the Pi-containing culture media was changed.

### Quantification of calcium deposition

Cells and tissues were decalcification with 0.6 N HCl at 4 °C for 24 h. The calcium content of the HCl supernatants was determined colorimetrically using Calcium assay kit (QuantiChromTM Calcium Assay Kit, BioAssay Systems, Hayward, WI, USA) according to the manufacturer's recommendations. Briefly, 5 μl of the samples was transferred to a 96-well plate. Working reagent (200 μl) was added and absorbance was then measured at 570 nm using a microplate ELISA reader (BioTek Instruments, Winooski, VT, USA). After decalcification, cells were washed three times with PBS and solubilized with 0.1 N NaOH/0.1% SDS. The protein content was measured with a BCA Protein Assay kit (Thermo Scientific Pierce, Rockford, IL, USA). The calcium content of RVSMCs or A10 cells was then normalized to the protein content, whereas that of the tissues was normalized to tissue dry weight.

For serum calcium measurement, blood samples were obtained from mice and kept on ice overnight and used for assay after centrifugation at 10,000 r.p.m. for 30 min. Serum calcium was assayed using the QuantiChrom calcium assay kit (BioAssay Systems).

### Determination of calcium deposition by calcium staining

Tissues to be stained for calcifications were collected and stored in 70% ethanol. Alizarin red staining was used to stain soft tissue calcifications. Each individual tissue sample was placed in 10 ml of alizarin working solution that contained 0.8% Alizarin red S in 0.5% KOH and was rotated for 24 h. The tissues were then removed from the Alizarin working solution, replaced in 10 ml of 0.05% KOH and rotated for another 24 h to remove any unbound stain from the tissues. A photograph was then taken to record the data.

For detection of mineralization of RVSMCs, von Kossa staining was performed^53^. In brief, cells were fixed with 10% formalin for 30 min at room temperature, washed with dH_2_O three times and then incubated with 5% silver nitrate for 30 min at room temperature. The cells were exposed to ultraviolet light for 2 h or overnight until colour development was complete. The silver nitrate solution was removed and the cells were washed with double distilled H_2_O and photographed by microscopy (Carl Zeiss, Jena, Germany).

### Quantitative real-time PCR

Total RNA from either cells or tissues was extracted using TRIzol Reagent (Invitrogen, Grand Island, NY, USA). The cDNA synthesis was generated using iScript cDNA Synthesis Kit (Bio-Rad Laboratories, Hercules, CA, USA) and analysed by real-time qPCR using a QuantiTech SYBR Green RT–PCR Master Mix (Qiagen, Valencia, CA, USA) and a Rotor gene Q (Qiagen, Hilden, Germany). All data were normalized to GAPDH. To rule out possible genomic DNA contamination, primers were designed to include an intervening intron. Amplimers were sequenced for confirmation. Primers for real-time qRT–PCR were as follows: RUNX2: 5′-CGCCTCACAAACAACCACAGAACC-3′, 5′-TGCTGCTGCTGTTGCTGCTGCTGC-3′; HDAC1: 5′- AGGGCACCAAGAGGAAAGTCTGTT-3′, 5′- TTCAGACTTCTTCGCATGGTGCAG-3′; HDAC2: 5′-GCCAACCCCGCTCTGCGATC-3′, 5′-GCCGCCTCCTTGACTGTACGC-3′; HDAC3: 5′-CAAGACCGTGGCGTATTTCTACGA-3′, 5′-GCCCAGTTGATGGCAATATCACAG-3′; HDAC4: 5′-GCATCCCTGTGTCATTTGGC-3′, 5′-CAGCGAGCTGTCCAGTTTCT-3′; HDAC5: 5′-TCCCGTCCGTCTGTCTGTTA-3′, 5′-GACATGCCATCCGACTCGTT-3′; HDAC6: 5′-CGAGTTCTTGCAGGCACCTA-3′, 5′-ATGCTCATAGCGGTGGATGG-3′; HDAC7: 5′-TGCTGGAGAAAGAAGAGATGATT-3′, 5′-GCAAGCCTGCTAGGAAGAGAT-3′; HDAC8: 5′-AGTTGGACGAGGGACTAGGG-3′, 5′-CTATTGGCGGGTTCCTCTGG-3′; HDAC9: 5′-ACCTACCGACAGTAGCAGCC-3′, 5′-TCCGGCCACTCCATCTGATT-3′; Chfr; 5′-AGGATTCCCGCATCGCCCCT-3′,5′-ACAGTTGCGGCCCCAGTAGC-3′; MDM2: 5′-GCGAGCGGAGACGGACACAC-3′, 5′-GGGCTCTGTGGCGCTTCCTC-3′; REN: 5′-TGCGACAACCCAGGAAGGCG-3′, 5′-CGGTCGGACGCTGCCTTCAG-3′; PIRH2: 5′-CCCTTCGCCCCGCAATCTGG-3′, 5′-CCAGCTTGGAAGCCACGCCT-3′; ATG3: 5′-ATTGCGACAGTCTCTCCGTG-3′, 5′-ACACCGCTTGTAGCATGGAA-3′; BARD1: 5′-GGTGTTACTGTCCCACGGAG-3′, 5′-CTTGGGCTTTCTGCTGAGGA-3′; FBXO4: 5′-CTTGAAAGCCAGCCGTCCTA-3′, 5′-CCTCGCTCTGTAGGCTGAAC-3′; FBXO32: 5′-CACTTCTCAGAGCGGCAGAT-3′, 5′-AGCAGCTCTCTGGGTTGTTG-3′; SIAH2: 5′-TAACCAATGCCGCCAGAAGT-3′, 5′-GCATCATCACCCAGTCCACA-3′; PELI1: 5′-TCTCAAGGCTCCTGACCAGT-3′ and 5′-TGTTGCTTATTGCCTTGGCG-3′.

### Immunoprecipitation and western blot analysis

Cells and tissues were collected with lysis buffer ((50 mM Tris (pH 8.0), 150 mM NaCl, 1 mM EDTA, 1% NP-40, 1 mM DTT, 1 mM phenylmethylsulfonyl fluoride, 1 mM Na_3_VO_4_ and 1 μg ml^−1^ each of leupeptin, pepstatin and aprotinin). One milligram of proteins was then immunoprecipitated overnight at 4 °C with the indicated antibody. After extensive washing with lysis buffer, the immunocomplexes were analysed by western blotting assay. The lysates were separated by SDS–polyacrylamide gel electrophoresis and transferred overnight at 100 mA on to a polyvinylidene difluoride membrane (Millipore, Bedford, MA, USA) and blocked with 5% skim milk in 1 × TBST. Membranes were incubated with the specific primary antibodies overnight at 4 °C. After three washes in 1 × TBST, membranes were incubated with horseradish peroxidase-linked secondary antibodies for 1 h at room temperature. Membranes were again washed three times in 1 × TBST, and protein bands were visualized by enhanced chemiluminescence using a Fuji LAS-3000 system (Fujifilm Life Science, Tokyo, Japan).

### GST pull-down assay

To investigate the direct interaction between MDM2 and HDAC1, an *in vitro* binding (GST pull-down) assay was performed. Both recombinant GST-HDAC1 and His-MDM2 proteins were obtained from *E. coli* transformed with *pGEX4T-HDAC1* and *pET28-MDM2*. Either GST or GST-HDAC1 was immobilized on a Sepharose 4B column. After the elution, His-MDM2 was applied to the column. The bound MDM2 was detected with western blot analysis using α-MDM2 antibody.

### Ubiquitination assay

To detect ubiquitinated HDAC1 proteins, cells in a 10-cm plate were transiently transfected with 4 μg *HA-ubiquitin* expression plasmids together with the indicated plasmid. Six hours before collecting, cells were treated with 25 μM of MG132. Samples were then lysed using a 1% NP-40 lysis buffer (50 mM Tris pH 7.5, 150 mM NaCl, 5 mM EDTA, 50 mM NaF, 1% NP-40, 5 mM NEM, 1 mM orthovanadate (Na_3_VO_4_) and 1 mM phenylmethylsulfonyl fluoride). The following procedures were identical to the immunoprecipitation assay except for the use of monoclonal anti-ubiquitin antibody.

### Tandem ubiquitin-binding entities assay

Commercially available kits (TUBE1 and TUBE2, LifeSensors, Malvern, PA, USA) were utilized according to the manufacturer's protocol with slight modification. For the TUBE assay, A10 cells were treated with Pi and MG132 and the cell lysates were used for the assay. Either GST or GST-TUBE2 was loaded on Sepharose 4B beads and the cell lysates were applied and assayed as in the conventional GST pull-down method. The precipitates were then separated on an SDS–polyacrylamide gel electrophoresis gel and transferred to the polyvinylidene difluoride membrane. The membrane was blocked with 5% bovine serum albumin and probed with either anti-HDAC1 or anti-Flag antibody.

### Induction of VC in mice

Six- to seven-week-old C57BL/6 male mice were used for VC induction by administration of VD_3_ as described previously^54^. VD_3_ (14.575 mg, 5 × 10^5^ IU kg^−1^ per day) in 70 μl of absolute ethanol was mixed with 500 μl Cremophor (Alkamuls EL-620, Sigma-Aldrich) for 15 min at room temperature, and this solution was then mixed with 6.2 ml sterilized water containing 250 mg of dextrose for an additional 15 min at room temperature. The mice were injected with a dose of VD_3_ (150 μl 25 g^−1^, 5 × 10^5^ IU kg^−1^ per day) subcutaneously for 3 days^55^.

To induction of VC in *ApoE* KO mice, 10-week-old *Apo*E KO male mice were fed a high-cholesterol diet for 10 weeks and were then fed a high-cholesterol plus calcium supplement diet for the following 7 weeks. VC was evaluated by Alizarin red S staining in transverse sections of the heart and aorta.

The atherosclerosis model was generated^56^. Subtotal occlusion of the left carotid artery was carried out in male *ApoE* KO mice^57^. A ventral midline incision was made in the neck. The left carotid artery was exposed and three of four caudal branches of the left carotid artery (left external carotid, internal carotid and occipital artery) were ligated with 7-0 silk suture under a surgical microscope, while the superior thyroid artery was lifted intact. The incision was then closed and the mice were monitored until recovery. After surgery, *ApoE* KO mice were fed Paigen's Atherogenic Rodent Diet (D12336; Research Diets, New Brunswick, NJ, USA) for 2 weeks until they were killed.

### Administration of MG132 or RG 7112 to mice

MG132 (2.5 mg kg^−1^ per day)^58^, RG 7112 (50 mg kg^−1^ per day)^59^, or appropriate vehicle or scramble was intraperitoneally administered to mice. All of these reagents were administered simultaneously with VD_3_ for the first 3 days and then the reagent alone at the same dose was administered for the next 6 days until the animals were killed.

### Smooth muscle-specific KO of *HDAC1* and *ApoE* KO mice

Floxed *HDAC1* was kindly provided by Professor Eric N. Olson of the University of Texas Southwestern Medical Center^60^. *Smooth muscle (SM)22α-cre* mice were purchased from Jackson Lab (cat.017491, Bar Harbor, ME, USA). Vascular smooth muscle-specific *HDAC1* KO mice were generated by breeding the floxed *HDAC1* mice with *SM22α-cre* mice. Deletion of *HDAC1* was confirmed by PCR-based genotyping^61^. The primers were as follows: *HDAC1* floxed: 5′-GCCTCTGCTTCCTTAGTGTTGG-3′, 5′-GAGCAAGGAAAGAGCACAAGCCTG; *SM22α cre*: 5′-ATTCTCCCACCGTCAGTACG-3′, 5′-CGTTTTCTGAGCATACCTGGA-3′. *ApoE* KO mice were purchased from Jung Ang Animal (Central Lab, Animal Inc., Seoul, Korea).

### Microcomputed tomography

Isolated heart and aorta samples were subjected to microcomputed tomography (Skyscan, Kontich, Belgium)^62^. The X-ray source was set at 50 kV and 200 μA with a pixel size of 17.09 μm. The exposure time was 1.2 s. Four-hundred fifty projections were acquired over an angular range of 180° (angular step of 0.4°). The tomographic acquired images were transformed into a sliced volumetric reconstruction by using the Nrecon program (Skyscan) and were analysed using three-dimensional Mimics imaging program (version 14.0, Materialise N.V., Leuven, Belgium).

### Human samples

Two different models of both intimal calcification and medial calcification were used. Atherosclerosis-associated VC samples were obtained from autopsied heart patients who died of myocardial infarction, whereas medial calcification samples were obtained from autopsied heart died of diabetes mellitus complications. Age-matched normal coronary artery was used for the control. For the atherosclerosis-associated intimal VC sample, paraffin-embedded left ventricle specimens and left anterior descending coronary artery were provided by the Department of Forensic Medicine, Chonnam National University Medical School. The samples had been obtained from eight individuals: four males with VC and four age-matched males. VC was diagnosed according to microscopic findings.

Human coronary arteries with intimal and medial calcification were obtained from autopsied hearts, and age-matched normal coronary arteries were used for the control. As samples were obtained from autopsied tissues, the written consent was not required. Exemption of subjects' written consent and approval for the use of the samples were obtained from the institutional review board of Chonnam National University Hospital (CNUH-2013-106).

### Histology

Mouse aorta and human coronary artery samples were fixed with 4% paraformaldehyde and embedded in paraffin. Cross-sections (5 μm) were stained with haematoxylin and eosin (H&E), Alizarin red S and immunohistochemistry with indicated primary antibody.

Quantification of histological images was performed after acquisition of the image by the use of iSolution FL (iMT technology, Canada) software. Two images from each sample were taken from the calcification adjacent area. The number of either HDAC1-positive or MDM2-positive nuclei were counted and divided by the total number of hematoxylin-positive nuclei.

### Statistical analysis

Data are represented as means±s.e.m. The data were analysed by the use of either the unpaired Student's *t-*test or one-way analysis of variance, which was followed by the Tukey honestly significant difference multiple-comparison *post hoc* test. When the Levene test for unequal variance was significant, Dunnett T3 was used as a *post hoc* test. Statistical analysis was performed with PASW Statistics 21 (SPSS, IBM Company, Chicago, IL).

Full blotting images corresponding to the immublottings shown in the main and Supplementary Figs are provided as Supplementary Fig. 9.

## Additional information

**Accession codes**: **cDNA microarray data deposition:** Gene expression data have been deposited in the GEO database under accession code GSE74755.

**How to cite this article:** Kwon, D.-H. *et al.* MDM2 E3 ligase-mediated ubiquitination and degradation of HDAC1 in vascular calcification. *Nat. Commun.* 7:10492 doi: 10.1038/ncomms10492 (2016).

## Supplementary Material

Supplementary Information Supplementary Figures 1-9
