# Supplementary material for: MDM2 E3 ligase-mediated ubiquitination and degradation of HDAC1 in vascular calcification
Source: Nat Commun. 2016 Feb 1;7:10492. doi: 10.1038/ncomms10492 (PMC4740400; doi:10.1038/ncomms10492)
Supplement: Supplementary Information — Supplementary Figures 1-9 [file ncomms10492-s1.pdf]

R3-NCOMMS-15-02003-T Kwon et al
Supplementary Figure-1

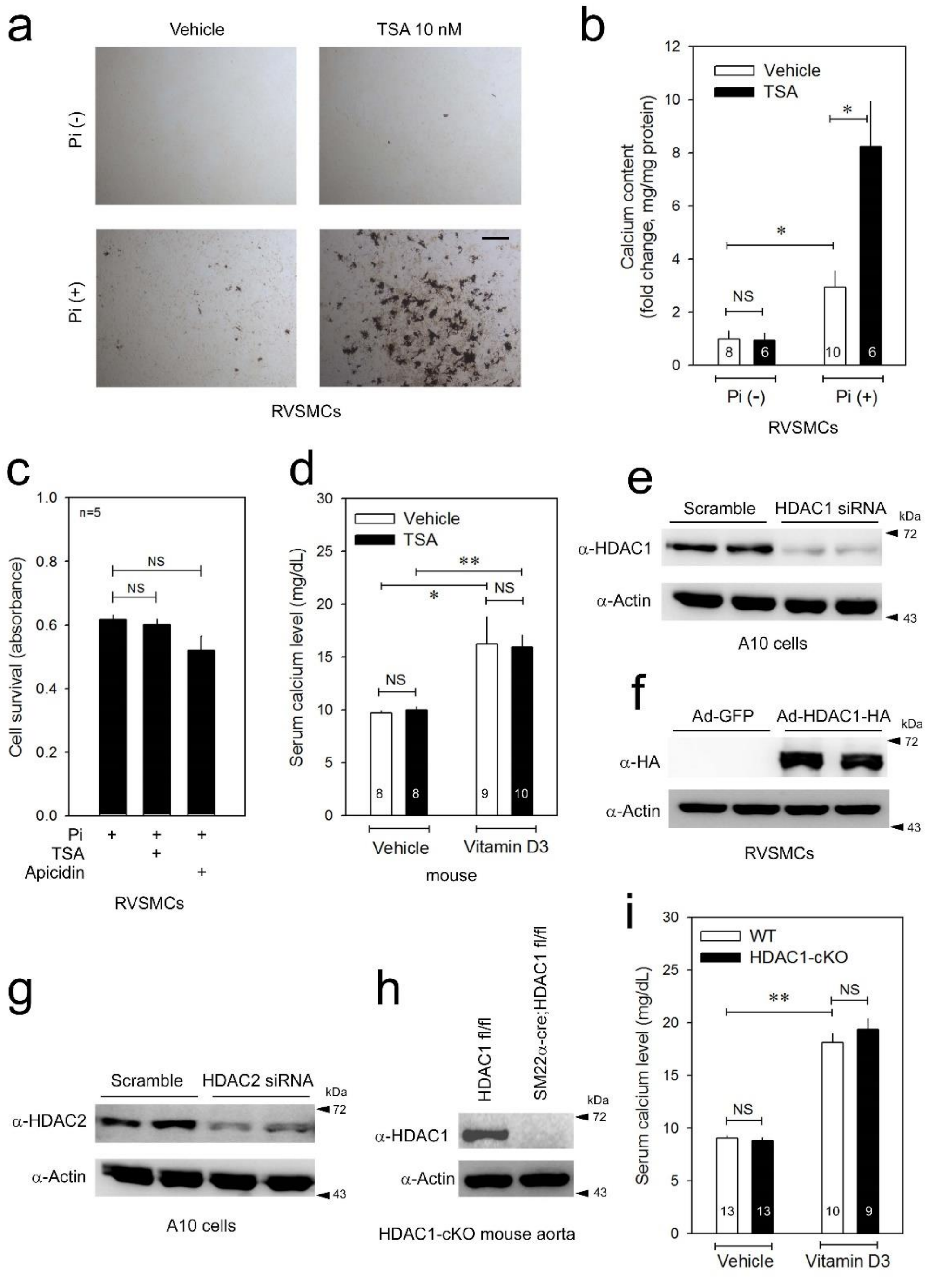

Supplementary Figure 1. TSA (10 nmol/L), non-class-selective HDAC inhibitor, potentiates vascular calcification (VC). (a) Von Kossa staining shows that TSA potentiated the Pi-induced VC. Scale bar, 100 μm. (b) Quantification results (n=6~8 from 2~3 sets). (c) Either TSA or apicidin (50 nM) did not affect RVSMC survival, as determined with MTT assay. Pi (2 mM) was treated for 6 days

(n=5 from 1 set). (d) TSA (0.6 mg kg^{-1}, i.p. for 9 days) did not affect serum calcium levels in the presence or absence of VC. VC was induced by VD_3, ($5x10^5$ IU kg^{-1} day, s.c. for initial 3 days) in 6~7-week-old C57BL/6 male mice (n=8~10 from 2 sets). (e) *HDAC1* siRNA successfully reduced the protein amount of HDAC1 in A10 cells, a rat vascular smooth muscle cell line. (f) Western blot analysis to show the successful Ad-HDAC1 infection. (g) Efficiency of knock-down of HDAC2 by *HDAC2* siRNA. (h) Generation of vascular smooth muscle-specific *HDAC1* deletion. Aorta samples were used for the detection of HDAC1 in either *HDAC1* $^{fl/fl}$ mice (WT) or *SM22*α*-cre;HDAC1* $^{fl/fl}$ mice (*HDAC1*-cKO). (i) Vascular smooth muscle-specific disruption of *HDAC1* did not affect the serum calcium level. VD_3 ($5x10^5$ IU kg^{-1} day, s.c. for initial 3 days) was administered to 6~8-week-old *HDAC1*-cKO male mice (n=9~13 from 3 sets). * $p<0.05$, ** $p<0.01$, NS: not significant. Numerals in bar graphs are the numbers of samples.

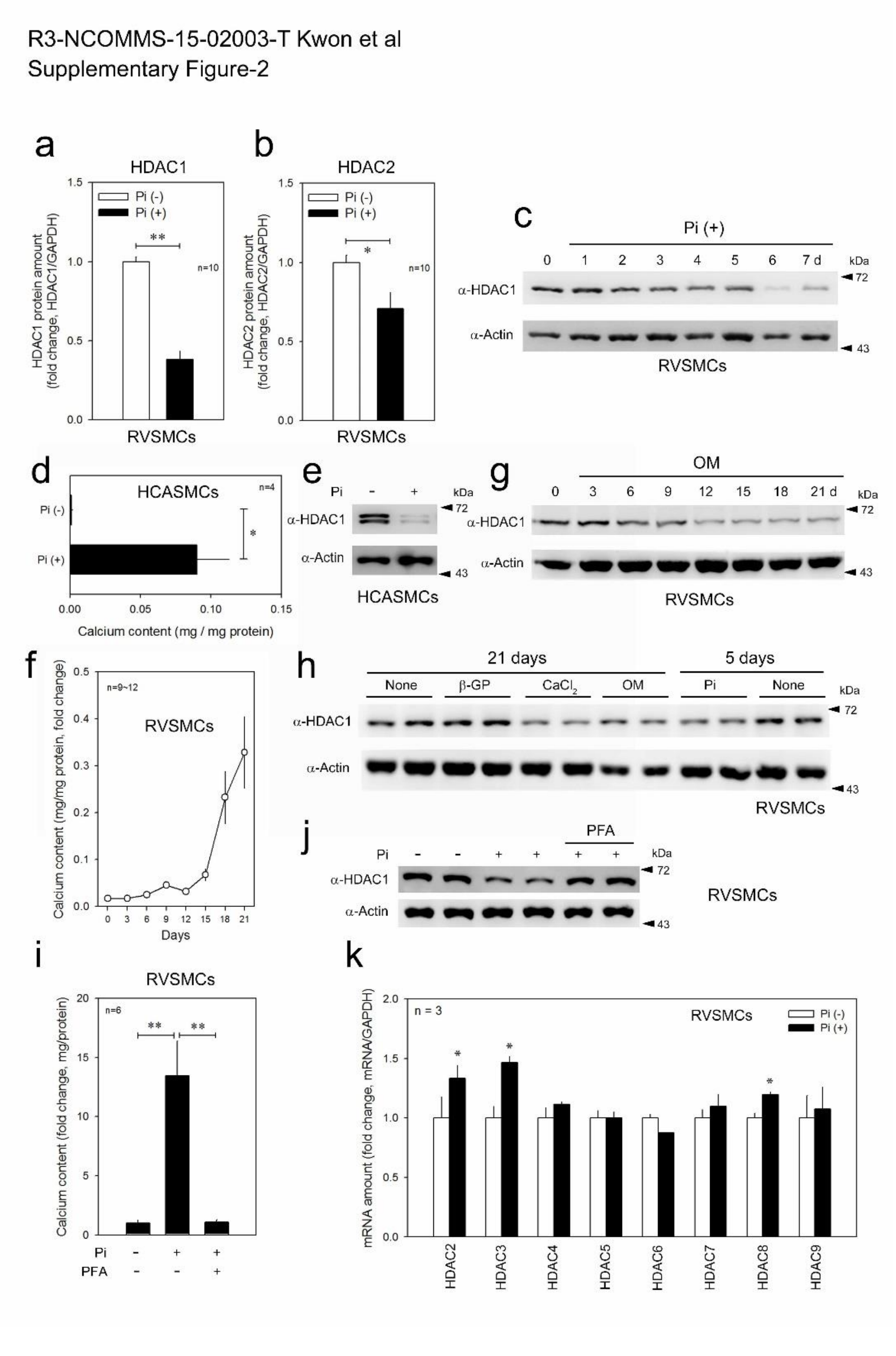

Supplementary Figure 2. Diverse calcification stresses reduce HDAC1 protein amounts, but not mRNA amounts *in vitro*. (a) Quantification of the Pi-induced reduction of HDAC1 protein amounts (n=10 from 6 sets). (b) Quantification result for HDAC2. Note that reduction of HDAC1 was greater than that of HDAC2 (n=10 from 6 sets). (c) Time course of HDAC1 protein reduction by Pi treatment in RVSMCs. (d) Pi-induced calcium deposition in human coronary artery smooth muscle cells (HCASMCs). Pi was treated for 6 days (n=4 from 2 sets). (e) Pi reduced the protein amount of

HDAC1 in HCASMCs. (f) Time course of osteogenic media (OM)-induced VC (n=9~12 from 2 sets). (g) OM also induced the reduction of HDAC1 protein amounts. (h) Reduction of HDAC1 protein amounts was reproduced by diverse calcification stresses such as $CaCl_2$ (8 mM), OM, and Pi (2 mM), but not by β-glycerophosphate (β-GP, 10 mM). (i) Phosphonoformic acid (PFA, 100 μM), an inhibitor of Pi transporter, blocked Pi-induced calcium deposition. Both PFA and Pi were treated for 6 days . (j) Effects of phosphonoformic acid (PFA, 100 μM), an inhibitor of Pi transporter on Pi-induced HDAC1 protein reduction. (k) Changes in *HDAC2-9* mRNA levels by Pi in RVSMCs. Each sample was measured in duplicate (n=3 from 2 sets). * $p<0.05$, ** $p<0.01$

R3-NCOMMS-15-02003-T Kwon et al
Supplementary Figure-3

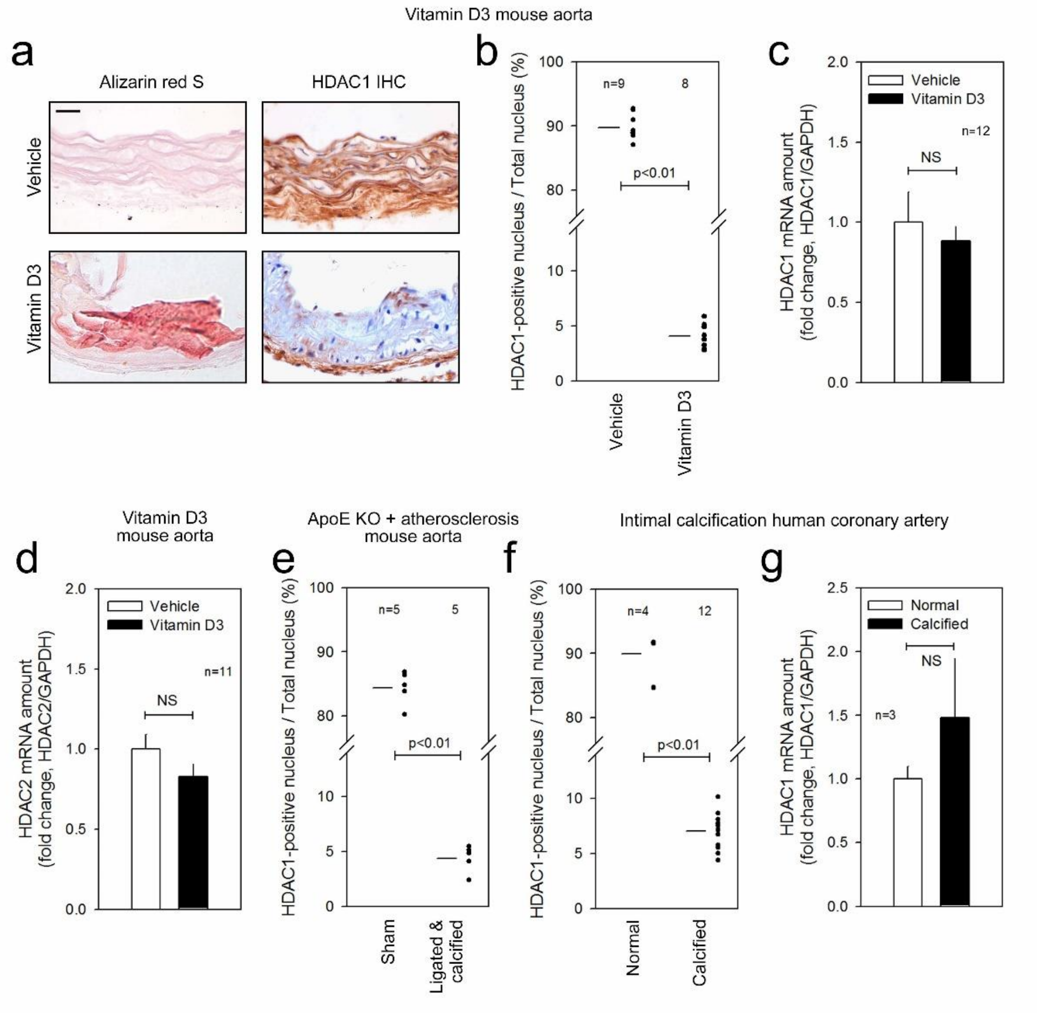

Supplementary Figure 3. Diverse calcification stresses reduce HDAC1 protein amounts, but not mRNA amounts *in vivo* models. Scale bar, 25 µm. (a) Immunohistochemical analysis showed that VD_3-administration induced reduction of HDAC1 at calcifying focus in blood vessels. (b) Quantification result of (a) (8~9 fields from 3 mouse-histology samples). (c) VD_3-administration did not affect mRNA levels of *HDAC1* (n=12 from 3 sets). (d) *HDAC2* mRNA level was not altered by VD_3-administration in mice (n=11 from 3 sets). (e) Quantification result of immunohistochemical analysis of HDAC1 expression in the aorta obtained from ApoE knockout mice with carotid artery ligation model (Fig. 3g, 5 fields from 2 mouse-histology samples). (f) Quantification result of immunohistochemical analysis of HDAC1 expression in the human intimal calcification model (Fig.

3h, 4~12 fields from 2~4 human-histology samples). (g) *HDAC1* mRNA level was not significantly altered in calcified human coronary artery samples (n=2~4, measured in duplicate). * p<0.05, ** p<0.01, NS: not significant.

R3-NCOMMS-15-02003-T Kwon et al
Supplementary Figure-4

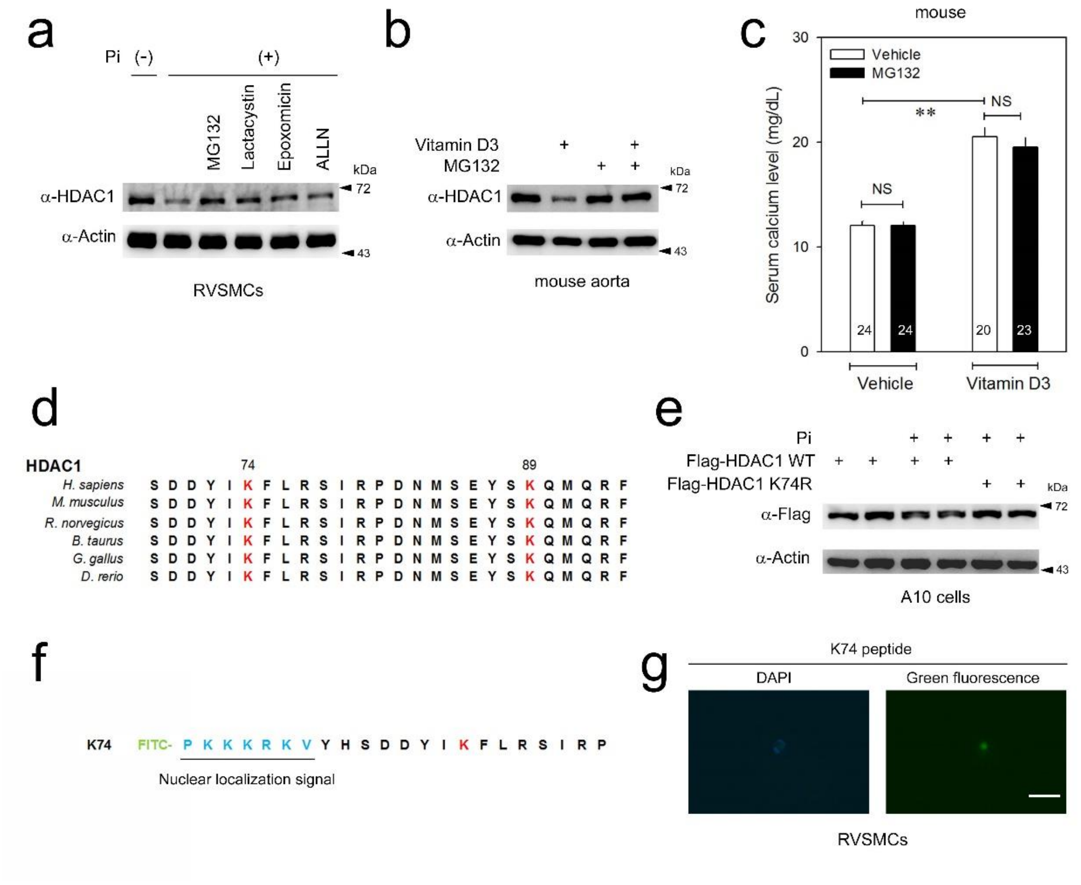

Supplementary Figure 4. HDAC1 K74 is ubiquitinated in VC. (a) HDAC1 degradation is proteasome-dependent. MG132 and alternative proteasome inhibitors such as lactacystin, epoxomicin, or *N*-[*N*-(*N*-Acetyl-L-leucyl)-L-leucyl]-L-norleucine (ALLN) successfully restored the Pi-induced reduction of HDAC1 protein amount in RVSMCs. MG132 (10 μM), lactacystin (10 μM), epoxomicin (1 μM), and ALLN (5 μM) were treated 8 hours before harvest. (b) Administration of MG132 to mice prevented the reduction of HDAC1 protein amount in the aorta. MG132 (2.5 mg kg^{-1} day, i.p.) was administered for 9 days, whereas VD_3 was treated for the first 3 days (n=20~24 from 4 sets). (c) MG132 did not affect serum calcium levels in the presence or absence of VC. (d) Sequence homology of HDAC1 in the various species. Note that all amino acids spanning two ubiquitination candidate

sites of K74 and K89 of HDAC1 are well-conserved. (e) Exogenous HDAC1 was also degraded by Pi. In A10 cells, mammalian expression vector of either *pBJ5.1-Flag-HDAC1wild type* (WT, 1 μg) or *pBJ5.1-Flag-HDAC1 K74R* (1 μg) was transfected with Lipofectamin LTX and Pi (2 mM) was treated for 3 days. Then, the anti-Flag antibody was used for Western blot analysis. Note that wild-type HDAC1 protein amount was reduced by Pi, whereas ubiquitination-resistant K74R mutant was not. (f) Structure of synthetic peptide spanning K74 (K74 decoy peptide). Fifteen amino acids (black) spanning K74 (red) were linked with the nuclear localization signal (bright blue color) and conjugated with FITC. (g) Successful delivery of K74 decoy peptide either to RVSMCs. K74 peptide was treated for 1 day at the concentration of 100 nM in RVSMCs. Scale bar, 100 μm.

R3-NCOMMS-15-02003-T Kwon et al
Supplementary Figure-5

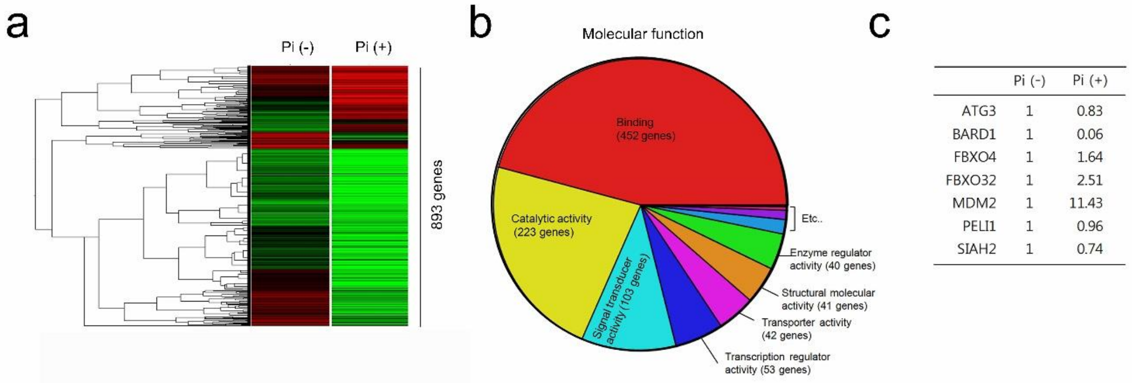

Supplementary Figure 5. cDNA microarray analysis to find E3 ligase. (a) cDNA microarray analysis to find the E3 ligase induced by Pi. Hierarchical clustering in the microarray analysis showed that the expression of 893 genes was changed in Pi-treated RVSMCs. (b) A Venn diagram was used to represent groupings of genes that showed expression level changes; the molecular function categories included binding, catalytic activity, and signal transducer activity. (c) Dysregulated genes that are expected to have E3 ligase function.

R3-NCOMMS-15-02003-T Kwon et al
Supplementary Figure-6

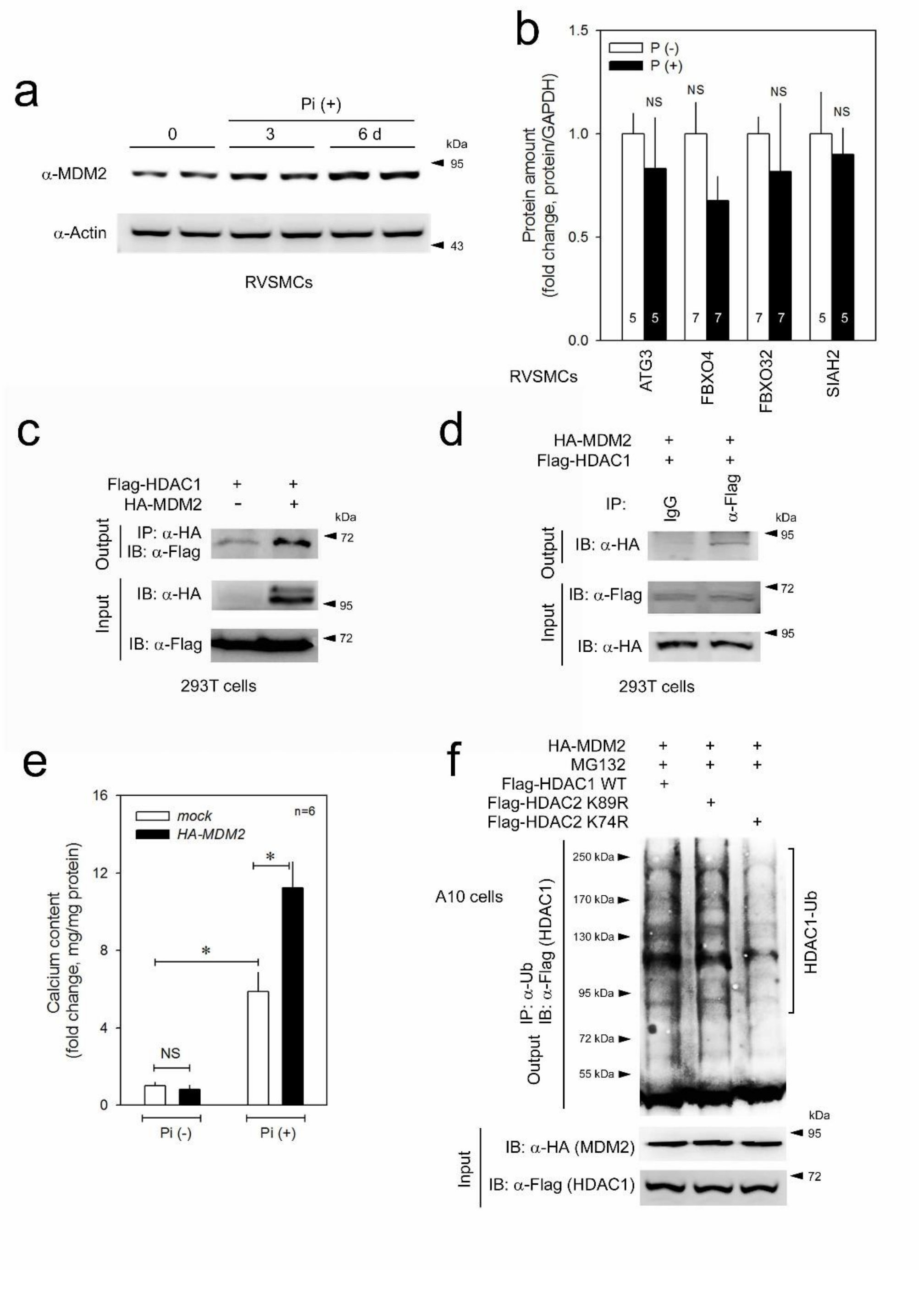

Supplementary Figure 6. MDM2 physically interacts with HDAC1 and potentiates calcium deposition. (a) Pi-treatment for 3 or 6 days increased MDM2 protein expression in RVSMC. (b) Changes in the protein amount of the other four E3 ligases that were significantly upregulated in

quantitative RT-PCR analysis (Fig. 5a) were further confirmed by Western blot analysis. Note that none of the other E3 ligases was significantly altered by Pi-treatment (n=5~7 from 3 sets). (c) Immunoprecipitation shows the physical interaction between exogenous HDAC1 and exogenous MDM2. Transfected MDM2 (anti-HA antibody) successfully recruited HDAC1 (Flag) in 293T cells. (d) Reverse immunoprecipitation to show that transfected HDAC1 pulled down MDM2 in 293T cells. (e) Transient transfection of *HA-MDM2* potentiated Pi-induced VC (n=6 from 2 sets). (f) MDM2-induced HDAC1 ubiquitination was attenuated in HDAC1 K74R, but not K89.

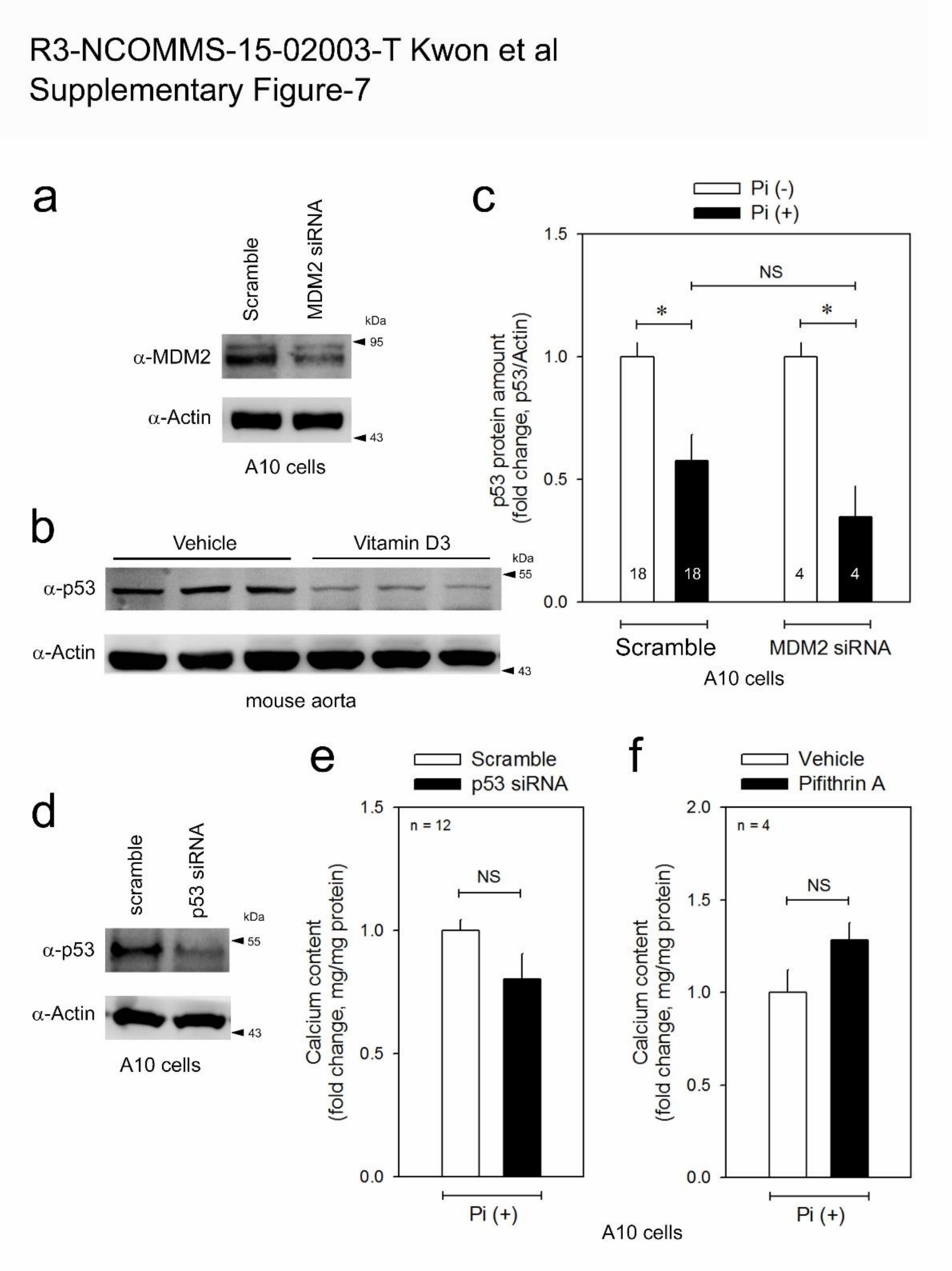

Supplementary Figure 7. p53 is not involved in Pi-induced VC. (a) *MDM2* siRNA (25 nM) successfully reduced the protein amounts of endogenous MDM2 in A10 cells. (b) Administration of VD_3 significantly reduced the protein amount of p53, an alternative target of MDM2, in aorta. (c) Pi also reduced p53 in RVSMCs. However, this reduction was not blunted by treatment with *MDM2* siRNA (n=4~18 from 2~5 experimental sets). (d) *p53* siRNA (50 nM) successfully reduced the protein

amounts of endogenous p53 in A10 cells. (e) *p53* siRNA failed to potentiate Pi-induced VC (n=12 from 3 experimental sets). (f) One micromolar pifithrin-α, a p53 inhibitor, failed to induce VC in RVSMCs (n=4 from 2 sets).

R3-NCOMMS-15-02003-T Kwon et al
Supplementary Figure-8

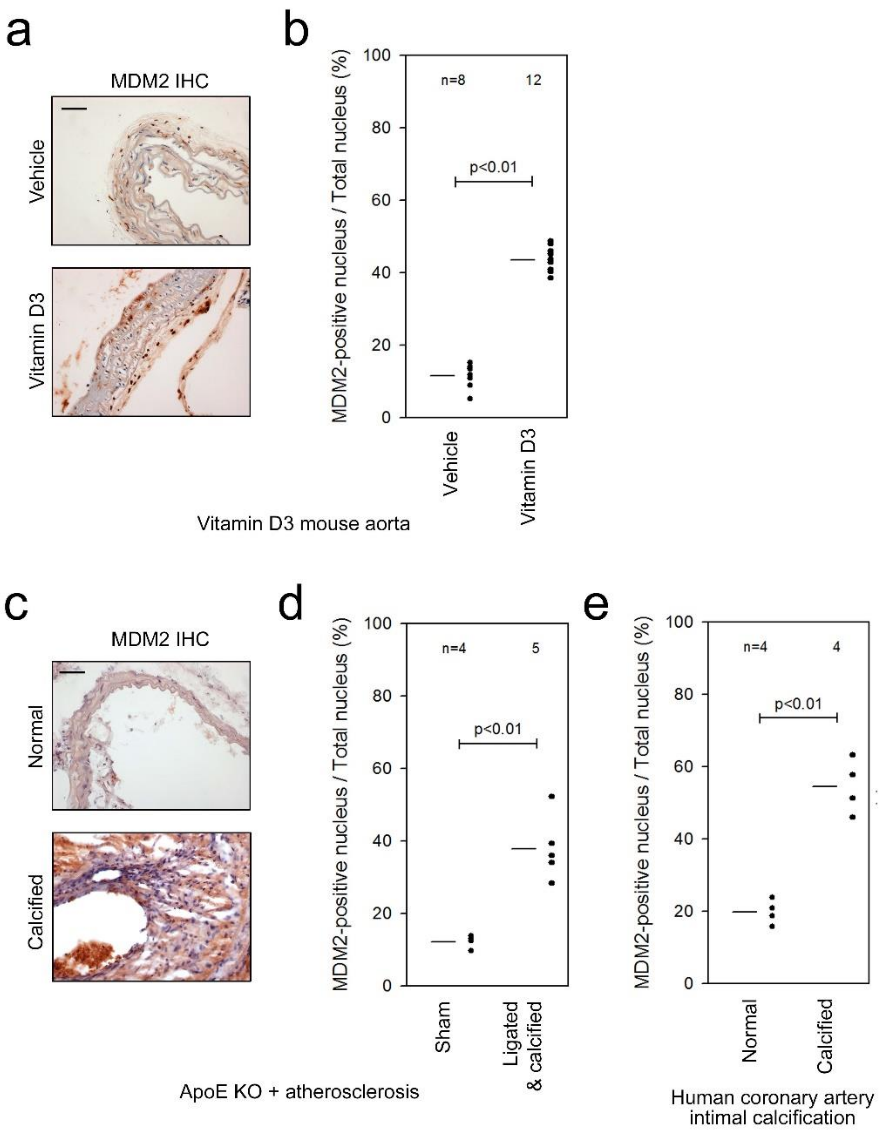

Supplementary Figure 8. Quantification of immunohistochemical analysis. (a) Immunohistochemical analysis showed the increase in MDM2 expression in VD_3-administered mice. Scale bar, 40 μm. (b) Quantification results of (a). Eight to 12 fields from 4 mouse-histology samples were examined. (c) MDM2 expression in the atherosclerosis-associated calcification model. ApoE KO mouse aorta was subjected to carotid artery ligation (ApoE+ligation). Scale bar, 40 μm. (d) Quantification results of (c). Four to 5 fields from 2 mouse-histology samples were measured. (e)

Quantification results obtained from the immunohistochemical analysis of MDM2 in human coronary intimal calcification samples (Fig. 7c). Four fields from 2 mouse-histology samples were measured.

Figure 3a

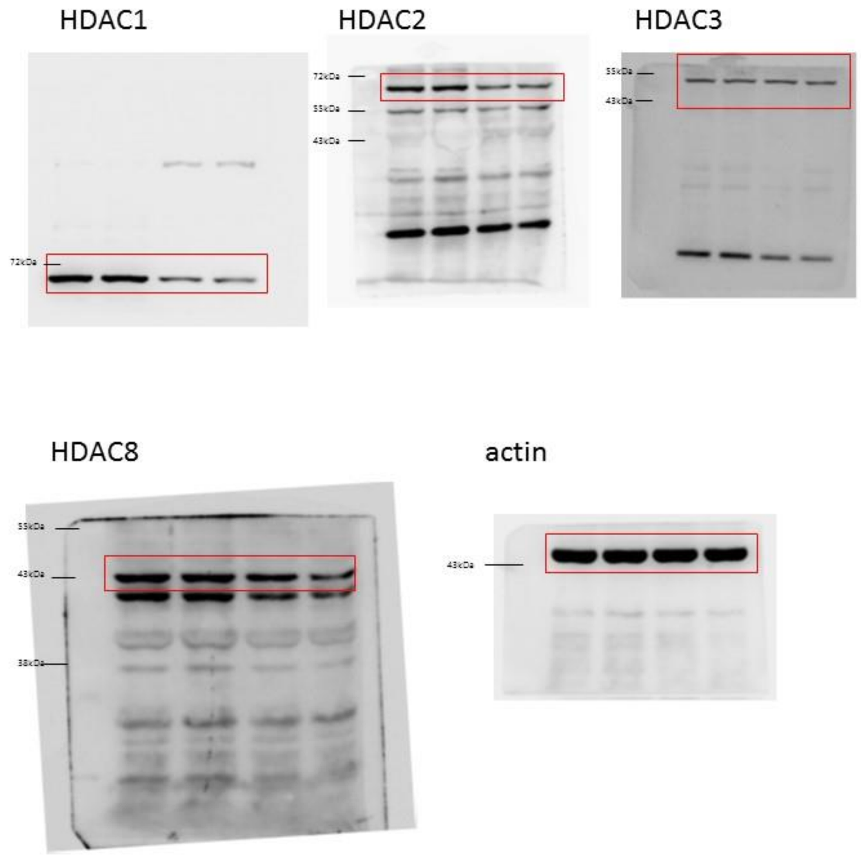

Supplementary Figure 9. Uncropped blots.

Figure 4b

HDAC1

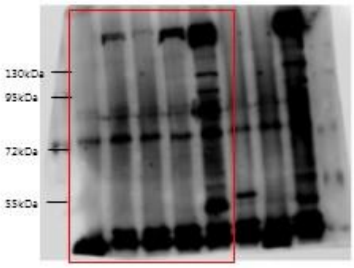

Flag

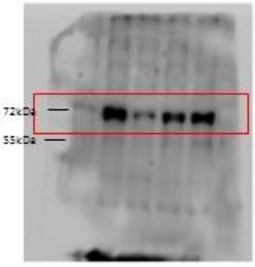

Figure 4c

HDAC1

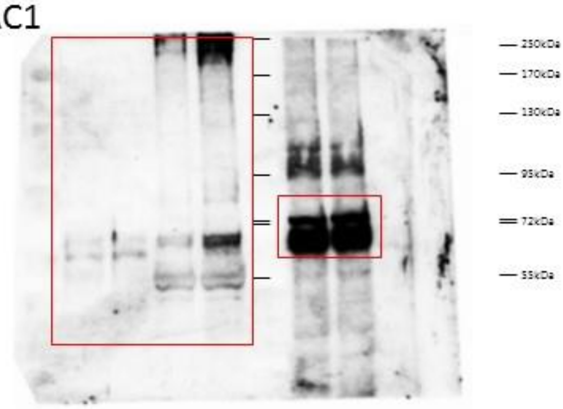

Figure 4h

HDAC1

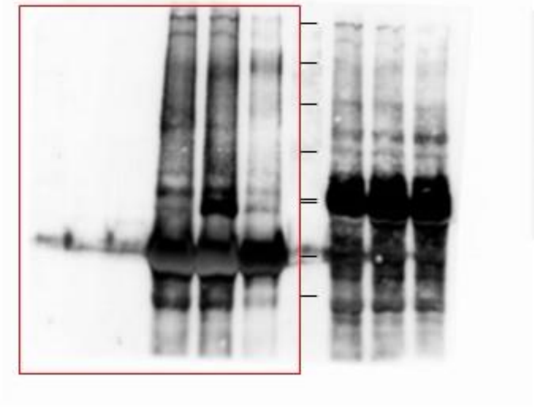

Flag

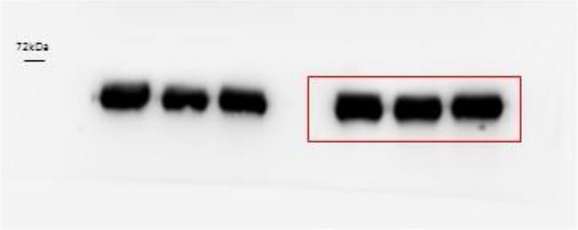

Supplementary Figure 9. Uncropped blots.

Figure 5d

MDM2

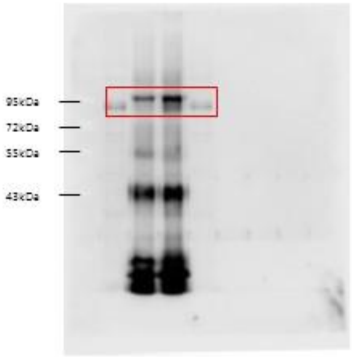

HDAC1

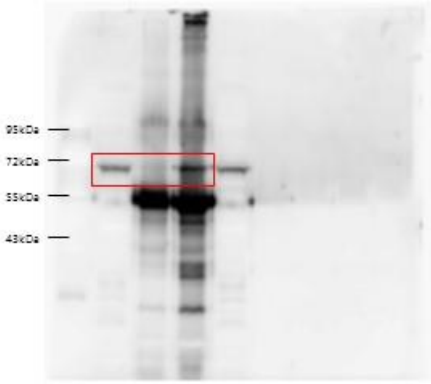

Figure 5e

Output: MDM2

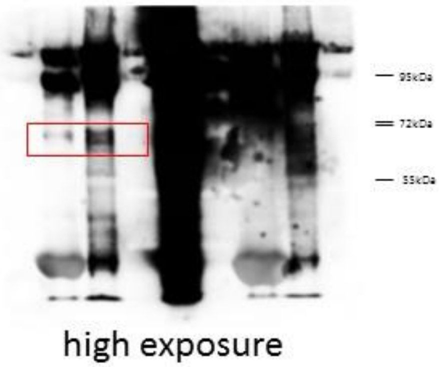

high exposure

Input: MDM2

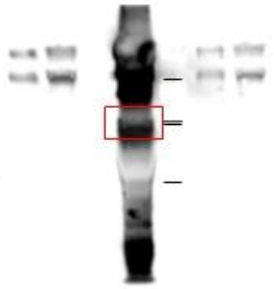

Low exposure

Coomassie blue staining

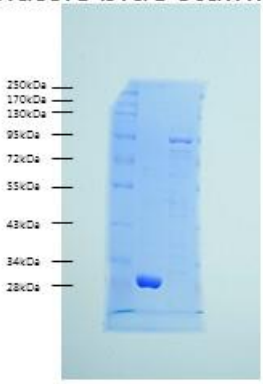

Figure 5f

HA

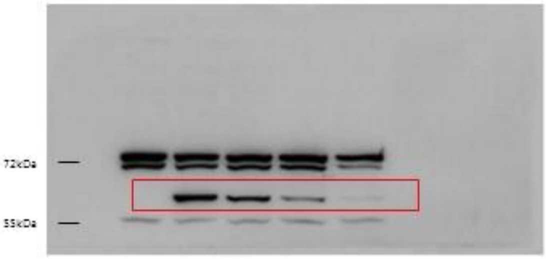

MDM2

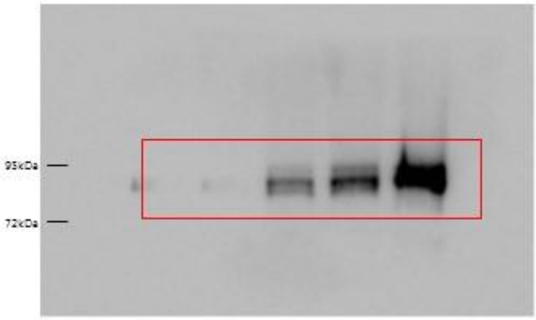

actin

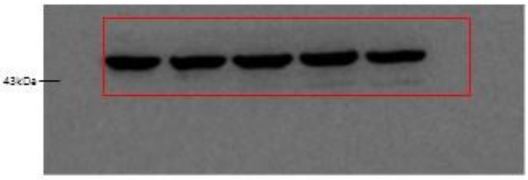

Supplementary Figure 9. Uncropped blots.

Figure 5g

Output, IB: Flag high exposure

Input, IB: Flag low exposure

Input: MDM2

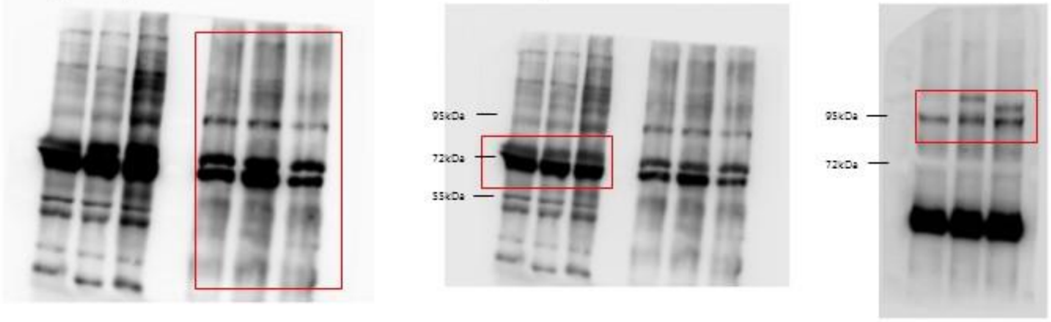

Figure 5h

Flag Input Output

Input: MDM2

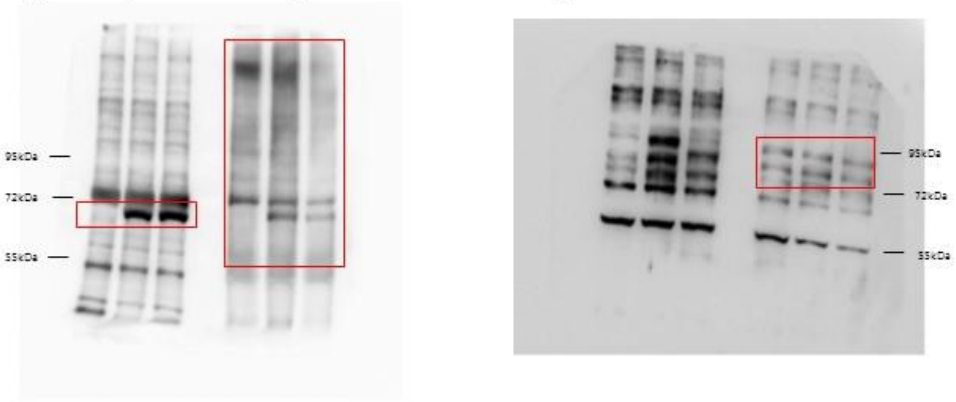

Supplementary Figure 9. Uncropped blots.

Figure 6c

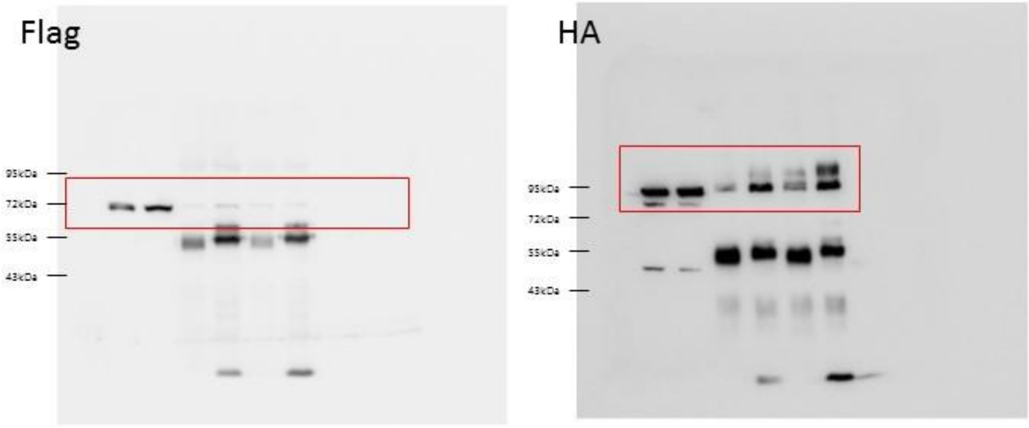

Figure 6d

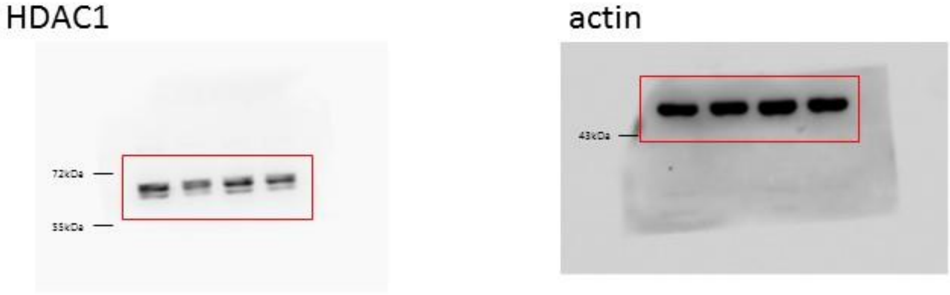

Supplementary Figure 9. Uncropped blots.

Figure 7f

HDAC1

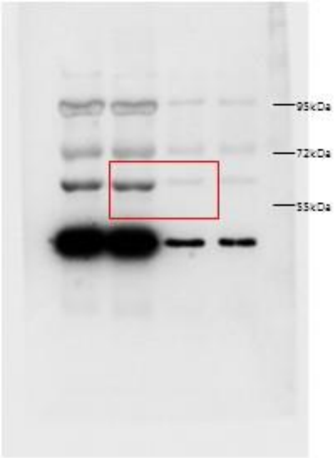

MDM2

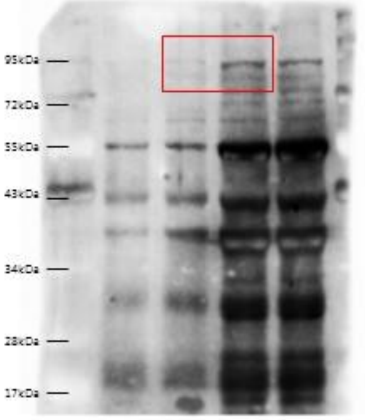

actin

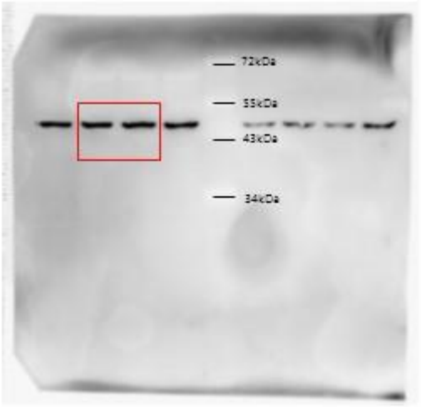

Figure 7g

HDAC1

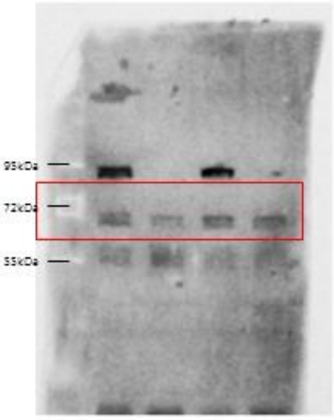

actin

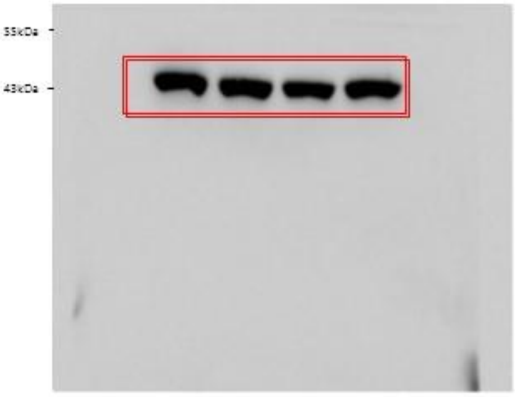

Supplementary Figure 9. Uncropped blots.

Supplementary Figure 1e

HDAC1

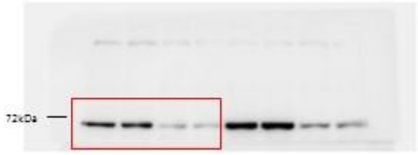

actin

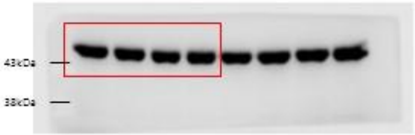

Supplementary Figure 1f

HDAC1

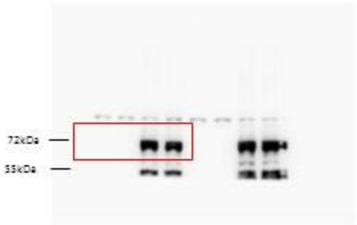

actin

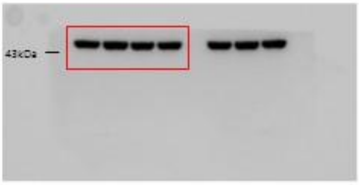

Supplementary Figure 1g

HDAC2

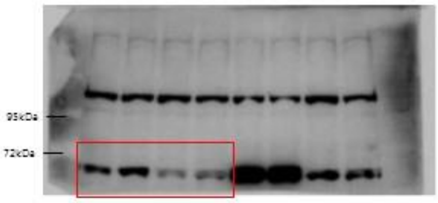

actin

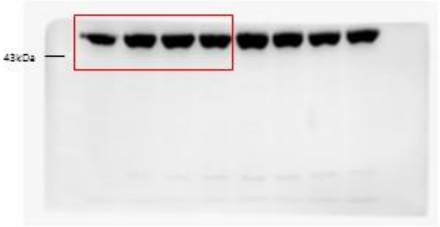

Supplementary Figure 1h

HDAC1

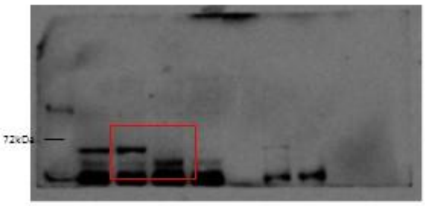

actin

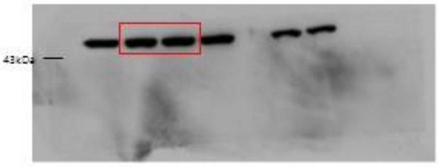

Supplementary Figure 9. Uncropped blots.

Supplementary Figure 2c

HDAC1

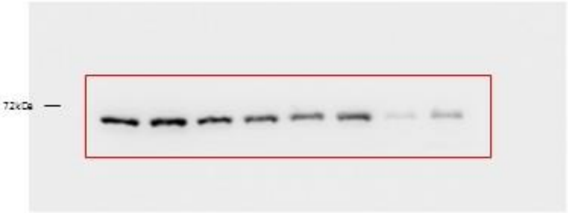

actin

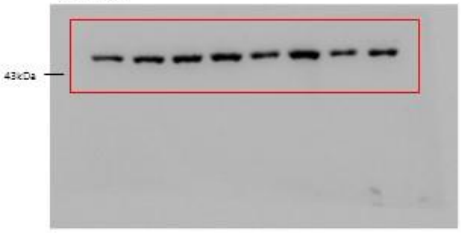

Supplementary Figure 2e

HDAC1

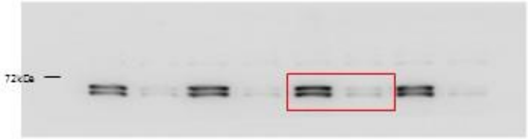

actin

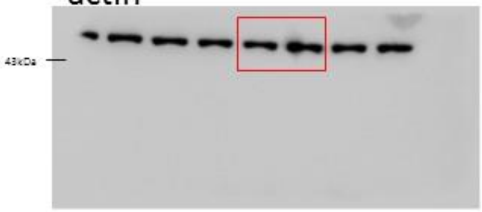

Supplementary Figure 2g

HDAC1

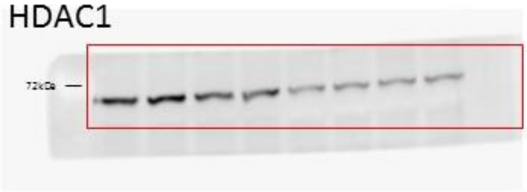

actin

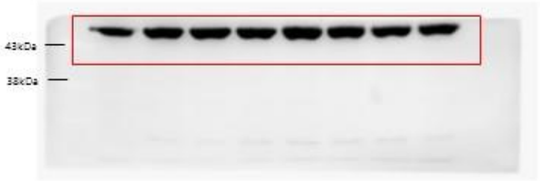

Supplementary Figure 2h

HDAC1

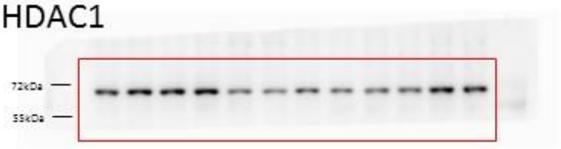

actin

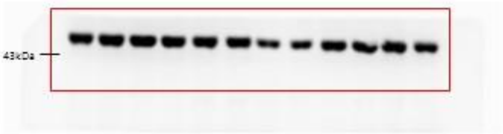

Supplementary Figure 2j

HDAC1

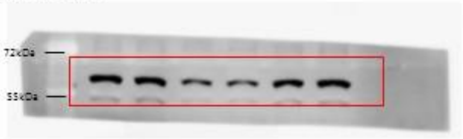

actin

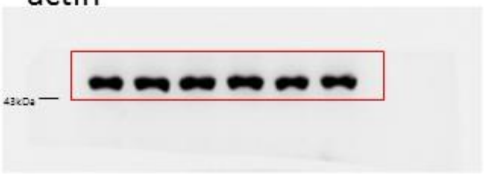

Supplementary Figure 9. Uncropped blots.

Supplementary Figure 4a

HDAC1

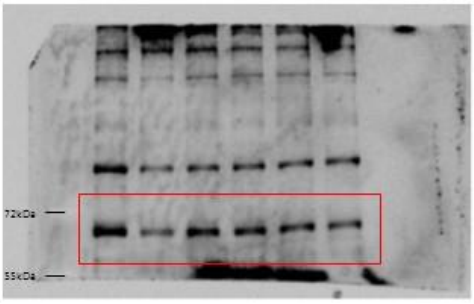

actin

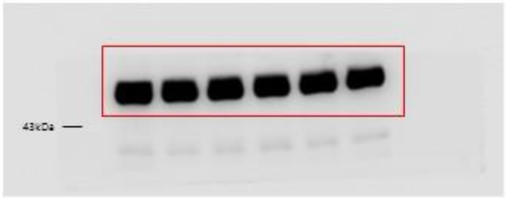

Supplementary Figure 4b

HDAC1

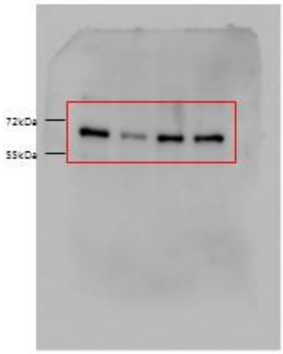

actin

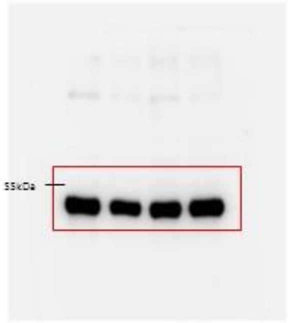

Supplementary Figure 4e

Flag

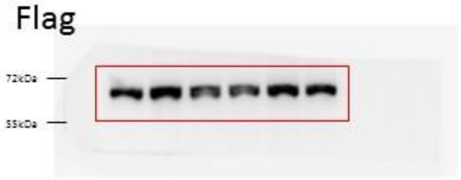

actin

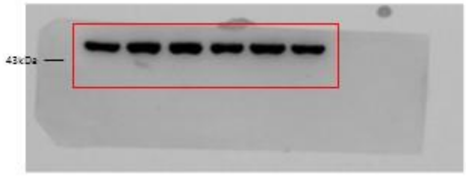

Supplementary Figure 9. Uncropped blots.

Supplementary Figure 6a

MDM2

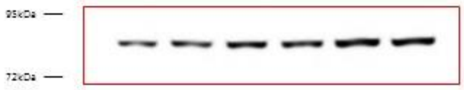

actin

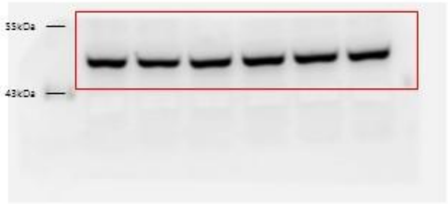

Supplementary Figure 6c

Flag

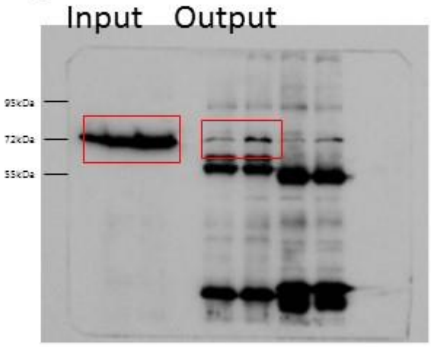

Input: HA

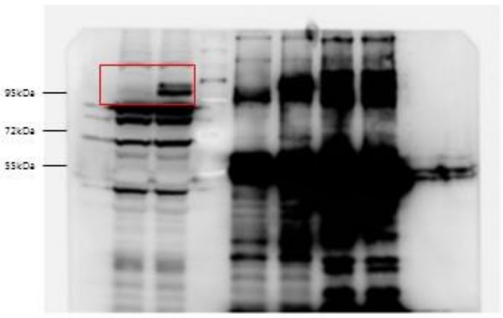

Supplementary Figure 6d

HA

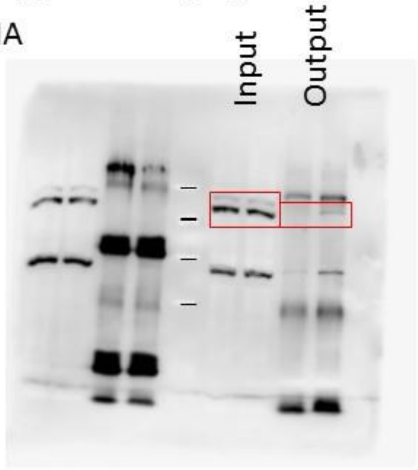

Input: Flag

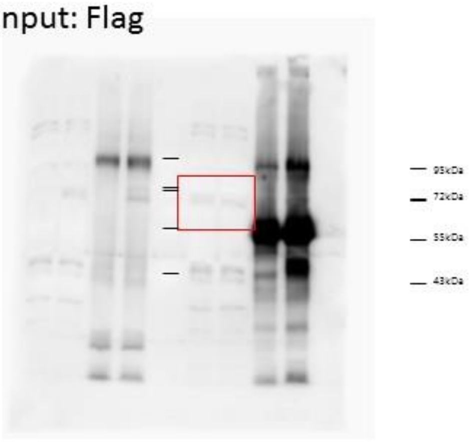

Supplementary Figure 9. Uncropped blots.

Supplementary Figure 6f

Output: Flag

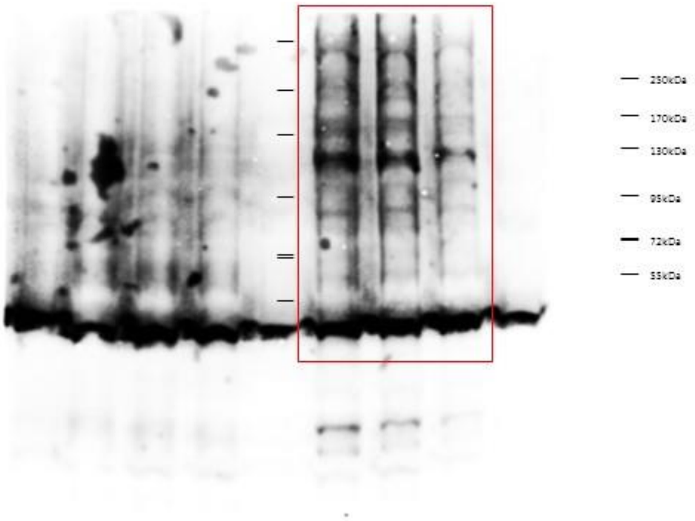

Input: HA

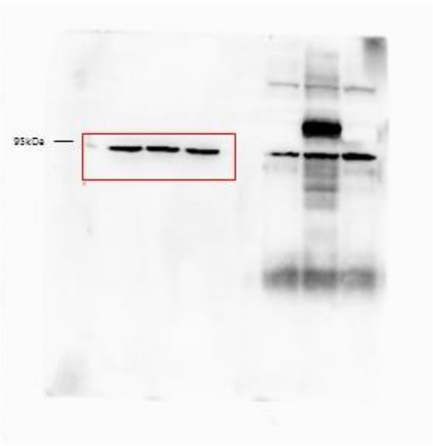

Input: Flag

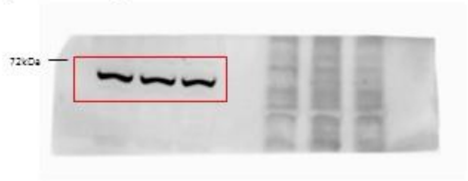

Supplementary Figure 9. Uncropped blots.

Supplementary Figure 7a

MDM2

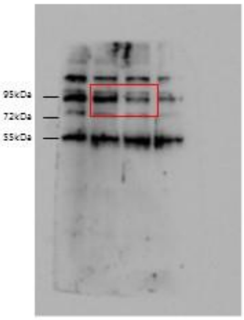

actin

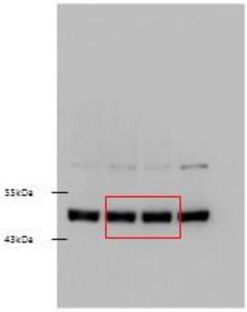

Supplementary Figure 7b

p53

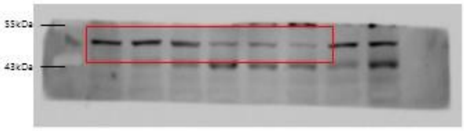

actin

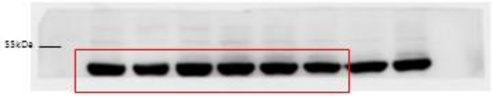

Supplementary Figure 7d

p53

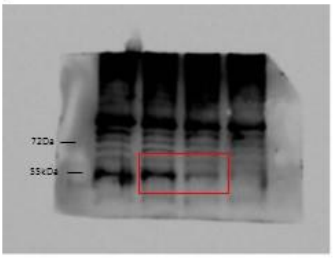

actin

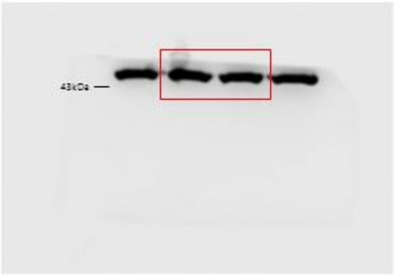

Supplementary Figure 9. Uncropped blots.
